# Supplementary material for: Global potential of sustainable single-cell protein based on variable renewable electricity
Source: Nat Commun. 2025 Feb 10;16:1496. doi: 10.1038/s41467-025-56364-1 (PMC11811160; doi:10.1038/s41467-025-56364-1)
Supplement: Supplementary file 1 — Supplementary Information [file 41467_2025_56364_MOESM1_ESM.pdf]

Supplementary Information for

Global potential of sustainable single-cell protein based on variable renewable electricity

Mahdi Fasihi [1,*], Fatemeh Jouzi [1], Petri Tervasmäki [2], Pasi Vainikka [2] and Christian Breyer [1]
[1] LUT University, Lappeenranta, Finland
[2] Solar Foods Oyj, Vantaa, Finland
* Corresponding author: mahdi.fasihi@lut.fi

Table of Content

Supplementary Note 1. History and projection of food protein consumption until 2050	2
Supplementary Note 2. Techno-economics data on Solar Foods' first small-scale and large-scale SCP core plant	3
Supplementary Note 3. Techno-economic comparison of Solar Foods' e-SCP plant and literature	5
Supplementary Note 4. Long-term development and techno-economics data on Solar Foods SCP core plant	10
Supplementary Note 5. Cost projection of alkaline water electrolyser	11
Supplementary Note 6. Energy and cost projection of solid sorbent Direct Air Capture	14
Supplementary Note 7. Supplementary results	16
Supplementary Note 8. Sensitivity analyses	27
Supplementary Note 9. Input data for optimisation	33
References	46

Supplementary Note 1. History and projection of food protein consumption until 2050

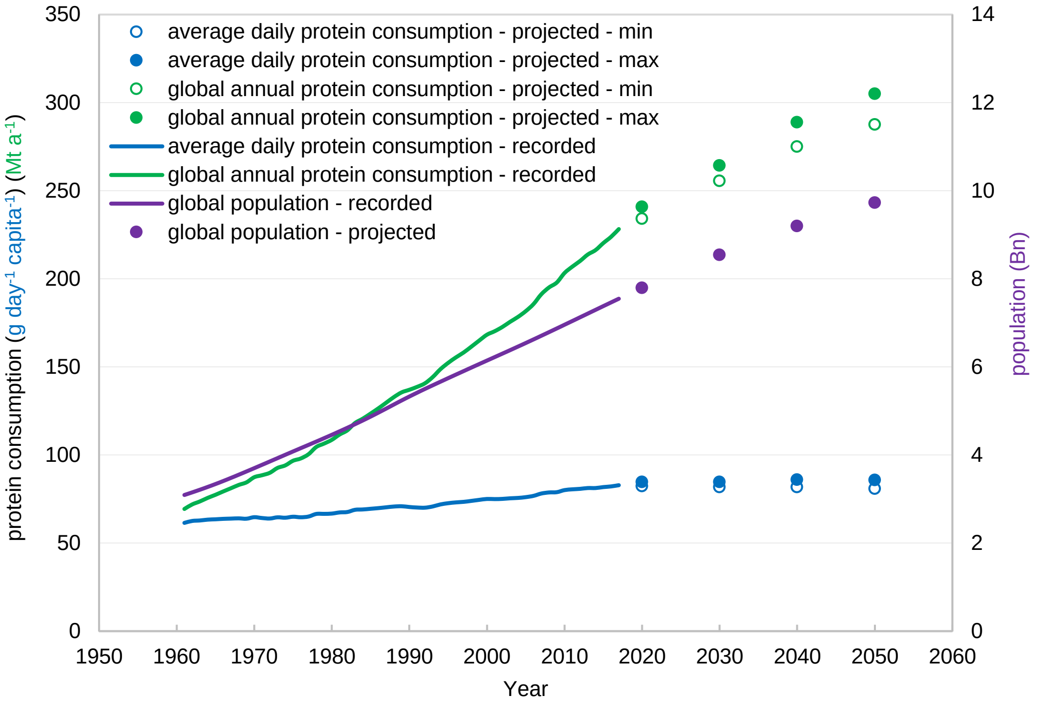

Supplementary Figure 1 | History and projections of global population and food protein consumption until 2050 by the Food and Agriculture Organization of the United Nations. In the past six decades, the global average daily protein consumption per capita has increased by about 35% from 61 g to 82.8 g and is projected to remain within 82.3−82.7 g until 2050. However, the global annual food protein consumption is affected by the increasing population and is projected to increase to 288−305 Mt by 2050[1]. Source data are provided as a Source Data file.

Supplementary Note 2. Techno-economics data on Solar Foods' first small-scale and large-scale SCP core plant

Throughout this study, the following naming style is used: The carbon-hydrogen-oxygen-nitrogen content of the desired biomass is called CHON content. The total mass of biomass, including CHON and additional mass originated from the consumed minerals, is defined as cell dry weight (CDW). The CDW produced in the fermentation tanks is referred to as CDW_{gross}. The total mass of CDW leaving the factory is less than CDW_{gross} due to losses in the separation of CDW from broth in the cell separation unit. The product leaving the factory is not fully dried and includes 5% moisture. The product leaving the factory (95% CDW and 5% moisture) is referred to as single cell protein or SCP.

Supplementary Table 1. Investment costs of the first small-scale and full-scale SCP core plant[a].

	Unit	Small-scale	Full-scale	
Number of 200 m^3 fermentation tanks		12	120	
Fermentation tanks total working capacity	m^3	2000	20,000	
Productivity	g_{CDW} L^{-1} h^{-1}	1.0	1.0	
Plant's net capacity	t_{SCP} h^{-1}	2.0526	20.526	
Investment costs				Scaling Factor
Media preparation, salt intake, CIP station	M€	7.7	54.5	0.85
Fermentation tank station	M€	35.5	251.3	0.85
Incubation and cell separation station	M€	15.2	107.6	0.85
Homogenizer, drum drying, packing station	M€	15.5	109.7	0.85
RO-ROP unit for water recovery	M€	2.2	15.6	0.85
Utilities	M€	13.2	93.4	0.85
Engineering, automation, commissioning	M€	11.7	23.3	0.30
Building and civil works	M€	12.6	107.2	0.93
Total direct and indirect costs (TD&IC)	M€	113.6	762.6	
Contingency (% of TD&IC)	M€	30	15	
Total Capital Investment	M€	147.7	877.0	
Capex	€ t^{-1}_{SCP} a	8995	5341	
Capex	€ kg^{-1}_{SCP} h	71,960	42,728	
Capex	€ $t^{-1}_{protein}$ a	14,567	8649	
Capex	€ $kg^{-1}_{protein}$ h	116,534	69,195	

Cost values in 2019 Euro. Abbreviations: Clean-in-Place (CIP), reverse osmosis and reverse osmosis polishing (RO-ROP).

[a] Land cost not included.

Supplementary Table 2. Annual fixed operational costs of the first small-scale and full-scale SCP core plant.

	Unit	Small-scale	Full-scale	
Fermentation tanks total working capacity	m^3	2000	20,000	
Plant's net capacity	$t_{SCP}\ h^{-1}$	2.0526	20.526	
Annual fixed operational costs				Scaling Factor
Maintenance (3% of TCI per year)	$k€\ a^{-1}$	4431	26,310	
Insurance (1% of TCI per year)	$k€\ a^{-1}$	1477	8770	
Personnel	$k€\ a^{-1}$	1855	5563	0.477
Other (outsourced services)	$k€\ a^{-1}$	502	2177	
cleaning	$k€\ a^{-1}$	14	70	0.70
legal consultation	$k€\ a^{-1}$	36	72	0.30
waste handling	$k€\ a^{-1}$	36	180	0.70
accounting	$k€\ a^{-1}$	36	109	0.48
laboratory analyses	$k€\ a^{-1}$	300	1 504	0.70
education, training	$k€\ a^{-1}$	80	242	0.48
Total annual fixed opex	$k€\ a^{-1}$	8265	26,310	
Total annual fixed opex	% of TCI	5.6	4.9	
Total annual fixed opex	$€\ t^{-1}_{SCP}\ a$	503	261	

Abbreviation: Total Capital Investment (TCI).

Supplementary Table 3. Energy and mass balance of the first small-scale and full-scale SCP core plant and outsourced variable operational costs.

	Demand			Price	Cost
	$kg\ kg^{-1}_{SCP}$	$kWh\ kg^{-1}_{SCP}$	$kWh_{th}\ kg^{-1}_{SCP}$	$€\ t^{-1}$	$€\ t^{-1}_{SCP}$
Onsite production				produced onsite	
H_2	0.4281				
O_2	1.989				
CO_2	1.824				
NH_3	0.148				
power – agitation		3.897			
power – auxiliary		4.577			
steam (10 bar) – drum dryer	5.25		3.354		
steam (4 bar) – pasteurisation and sterilisation	3.61		2.303		
chilled water	1686				
Outsourced					
minerals[2]	0.0543			270	14.65
process water [a]	10.6			3	31.88
wastewater [b]	14.0			2	28.05
exhaust gas management [c]	0.0425			10	0.43
Total outsourced variable operational cost					75.0

[a] For media preparation, steam boiler, and ammonia solution. The water required for H_2 production is separately accounted for in variable opex of water electrolyser.

[b] From dryer, cell separation, and steam consumption in pasteurisation and sterilisation.

[c] Including 10% H_2, 37% O_2, 50% CO_2, and 3% H_2O.

Supplementary Note 3. Techno-economic comparison of Solar Foods' e-SCP plant and literature

3.1. Stoichiometric mass balance

Ishizaki and Tanaka (1990)[3] performed a lab-scale experiment on the production of *Cupriavidus necator, Alcaligenes eutrophus* ATCC 17697^T. The overall stoichiometry of this biomass production by hydrogen oxidisation in a closed looped batch culture (500 ml reactor with 100 ml working volume) is provided in supplementary equation (1).

This equation could be normalised per mole of carbon, as shown in supplementary equation (2). The molar mass of $CH_{1.74}O_{0.46}N_{0.19}$ is 23.74 g mol^{-1}.

Another similar study by Bongers (1970)[4] reported the stoichiometry provided in supplementary equation (3) considering only C, H and O atoms in the biomass. The stoichiometry provided by Bongers (1970)[3] requires about 15% less H_2 and 20% less O_2 for fixation of 1 mole of CO_2, compared to the stoichiometry provided by Ishizaki and Tanaka (1990)[4].

Ishizaki and Tanaka (1990)[3] reported nearly 100% utilisation of feed gases through recirculation of unreacted feed gases. The study also concluded that the conversion rate of feed gases is affected by the applied nutrients, culture environment, and oxygen concentration. While a lower concentration of oxygen increases the growth rate, it simultaneously reduces the cell mass concentration[3].

As discussed by both Bongers (1970)[4] and Ishizaki and Tanaka (1990)[3], the energy efficiency of the CO_2 fixation may vary within the same organism due to environmental factors. For example, a limitation in CO_2 supply would result in excess H_2 oxidation without corresponding CO_2 fixation resulting in less efficient growth stoichiometry.

The literature on the techno-economic assessment of e-SCP production[5–8] is mainly based on the *Cupriavidus necator* ($CH_{1.74}O_{0.46}N_{0.19}$) formation stoichiometry by Ishizaki and Tanaka (1990)[3].

Here we also adapt the stoichiometry from Ishizaki and Tanaka (1990)[3] for the CHON composition of Solar Foods' product microbe. The microbe is *Xanthobacter* sp. SoF1 as described in Klinzing et al. (2024)[9] and Holmström and Pitkänen (2021)[10]. Approximation of the stoichiometry of the reaction is presented in supplementary equation (4).

The CHON composition of the biomass considered by Solar Foods ($CH_{1.7}O_{0.37}N_{0.21}$) has a molar mass of 22.56 gram per mol g mol^{-1}. In addition, the consumed minerals make up 5% of the CDW, increasing the biomass molecular weight to about 23.75 g mol^{-1}. The overall stoichiometric demands per 1 mole of carbon remain very close to the values presented in supplementary equation (2). Only ammonia demand is 13% higher due to higher protein content whereas the difference for hydrogen and oxygen is negligible. However, given the differences in the molar mass of CHON contents, the overall stoichiometric demand of CO_2, H_2, O_2, and NH_3 per 1 kg of CHON content in Solar Foods system are respectively 5.2, 4.1, 4.0, and 18.9% higher than those in Ishizaki and Tanaka (1990)[3].

Liu et al. (2016)[11] developed lab-scale hybrid water splitting–biosynthetic systems with 100- and 1000-ml batch reactors for production of Cupriavidus necator. The reported stoichiometry is provided in supplementary equation (5), which has higher hydrogen, oxygen, and nitrogen content per mole of assimilated carbon in the produced biomass, compared to the two previously mentioned studies. The molar mass of $CH_{1.77}O_{0.49}N_{0.24}$ is 24.97 g mol^{-1}. The values reported by Liu et al. (2016)[11] are used by Sillman et al. (2019)[12] and Sillman et al. (2020)[13] for life cycle assessment of Power-to-SCP system with in-situ water electrolysis. The same biomass composition was also considered earlier in a techno-economic assessment of e-SCP by Leger et al. (2021)[14].

$$4.09\ CO_2\ (g) + 21.36\ H_2\ (g) + 6.21\ O_2\ (g) + 0.76\ NH_3\ (aq) \rightarrow C_{4.09}H_{7.13}O_{1.89}N_{0.76}\ (s) + 18.70\ H_2O \quad (1)$$

$$CO_2\ (g) + 5.22\ H_2\ (g) + 1.52\ O_2\ (g) + 0.19\ NH_3\ (aq) \rightarrow CH_{1.74}O_{0.46}N_{0.19}\ (s) + 4.56\ H_2O\ (l) \quad (2)$$

$$CO_2\ (g) + 4.44\ H_2\ (g) + 1.22\ O_2\ (g) \rightarrow CH_2O\ (s) + 3.44\ H_2O\ (l) \quad (3)$$

$$CO_2\ (g) + 5.165\ H_2\ (g) + 1.5\ O_2\ (g) + 0.21\ NH_3\ (aq) \rightarrow CH_{1.7}O_{0.37}N_{0.21}\ (s) + 4.63\ H_2O\ (l) \quad (4)$$

$$\text{electricity} + CO_2\ (g) + 0.525\ H_2O\ (l) + 0.24\ NH_3\ (aq) \rightarrow CH_{1.77}O_{0.49}N_{0.24}\ (s) + 1.02\ O_2\ (g) \quad (5)$$

Liu et al. (2016)[11] did not specify the overall hydrogen production and its efficiency. However, they reported on an average electricity-to-biomass efficiency of 54% and 47% for the systems with 100- and 1000-mL bioreactor volume, respectively. Leger et al. (2021)[14], on the other hand, considered a range of 0.43−0.47 kg_{H2} demand per kg_{CHON}.

The respective stochiometric and empirical feed gases required per kg of CHON formation and delivered SCP for the above-mentioned systems are provided in Supplementary Table 4. The empirical demand for gaseous feedstock per kg of delivered SCP or its protein content are affected by the utilisation rate of gaseous feedstock, minerals content of CDW, CDW loss in the post-processing system, and the moisture content of the delivered SCP, which are mostly not accounted for in the available literature.

3.2. Empirical mass balance

In 2015, the Belgian institute InnovationNetwork published a report[15] on the feasibility of farm-scale Power-to-Protein in collaboration with Avecom, a company with experience in production of microbiomes. The study is based on the stoichiometry by Ishizaki and Tanaka (1990)[3] and a conservative 80% assimilation of feed gases, leading to production of 0.23 kg of CDW_{gross} per kg of chemical oxygen demand (COD). However, the study acknowledges that the yield rate could be increased to 0.25−0.3 by improving the culture environment. A yield of 0.3 kg_{CDW} per kg_{COD} is not achievable even with 100% utilisation rate of feed gases based on the referenced stoichiometry. This reflects the impact of the bioreactor environment and oxygen factor in improving the stoichiometry of CDW production.

In 2016, Matassa et al.[16] (affiliated with Avecom) reported on a series of lab-scale experiments for autotrophic hydrogen-oxidizing bacteria production. The experiments considered open culture by use of aerobic sludge as the source of nitrogen, and compressed air as the source of oxygen. The study was performed in 3-litre sequence batch and continuous reactors. According to this study, the average productivity of the system with continuous reactor (0.375 g_{CDW} L^{-1} h^{-1} Sulfuricurvum spp.) was significantly higher than that with sequence batch reactor (0.078 g_{CDW} L^{-1} h^{-1} mixed culture).

Supplementary Table 4. Comparison of stochiometric and empirical mass balance of feed gases from literature and this study.

	Feed gases				CHON content of dry matter	Feed gases utilisation rate	Protein content	Comment	Reference
	CO_2	H_2	O_2	NH_3		%	% of CDW		
	stoichiometric balance								
kg kg^{-1}_{CHON}	1.854	0.440	2.047	0.133	$C_1H_{1.74}O_{0.46}N_{0.19}$	100		closed cycle	Ishizaki and Tanaka[3]
kg kg^{-1}_{CHON}	1.950	0.458	2.128	0.158	$C_1H_{1.70}O_{0.37}N_{0.21}$			closed cycle	this study (Solar Foods)
kg kg^{-1}_{CHON}	1.762	0.378 kg_{H2O}		0.163	$C_1H_{1.77}O_{0.49}N_{0.24}$			in-situ water electrolysis	Liu et al.[17]
	empirical balance								
kg kg^{-1}_{SCP}	5.710	1.712	air					batch open culture	Matassa et al.[16]
kg kg^{-1}_{SCP}	2.046	0.446	air					continuous open culture	Matassa et al.[16]
kg kg^{-1}_{SCP}	2.316	0.550	2.047	0.134		80		open system	InnNet[15] – reported
kg kg^{-1}_{SCP}	2.317	0.550	2.558	0.166		80		open system	InnNet[15] – recal.
kg kg^{-1}_{SCP}	1.824	0.428	1.989	0.148		99	65	closed cycle	Solar Foods
kg $kg^{-1}_{protein}$	9.361	2.807					61	batch open culture	Matassa et al.[16]
kg $kg^{-1}_{protein}$	2.882	0.628					71	continuous open culture	Matassa et al.[16]
kg $kg^{-1}_{protein}$	3.309	0.786	2.924	0.238		80	70	open system	InnNet[15] – reported
kg $kg^{-1}_{protein}$	3.296	0.782	3.639	0.246		80	70	open system	InnNet[15] – recal.
kg $kg^{-1}_{protein}$	2.954	0.693	3.221	0.240		99	65	closed cycle	this study (Solar Foods)

Abbreviations: carbon-hydrogen-oxygen-nitrogen (CHON), recalculated (recal.), and InnovationNetwork (InnNet).

In 2019, the Dutch KWR Water Research Institute in cooperation with Avecom and others reported on a pilot design for an aerobic hydrogenotrophic fermentation bioreactor with 580 litres volume and a potential SCP production capacity of 1.7 kg per day[18]. However, the intended volumetric production capacity was not achieved due to insufficient hydrogen mass transfer in the new reactor system. The new reactor design was open at the top for discharge of unabsorbed hydrogen to the environment for safety considerations[18].

3.3. Economic data

Economic data on electricity-driven SCP is scarce. A recent (2023) review article on SCP pathways concluded that unavailability of economic data on novel SCP pathways, such as e-SCP, has stalled their detail techno-economic analysis[19]. To the best of our knowledge, there exists five scientific articles covering the economics of e-SCP, which we explain below. Table 1 provides a summary of the techno-economic data from the reviewed literature, and Supplementary Fig. 2 visualises the comparison of key values for techno-economic evaluations. The text below mainly uses the reported values in their original unit, while the respective values in the table are adjusted to unified units.

Pikaar et al. (2018)[8] investigated the economics of e-SCP production by a 25,000 t_{CDW} a^{-1} plant, by assuming a capital investment of 75 mUSD, 25 years technical and financial lifetime, as well as 5% interest rate. They considered 80% utilisation rate of feed gases based on the stoichiometry of SCP formation by Ishizaki and Tanaka (1990)[3]. However, the reported H_2 demand of 0.46 kg_{H2} kg^{-1}_{CDW} compared to stoichiometric demand of kg_{H2} kg^{-1}_{CHON} represents an H_2 utilisation rate of 95.7%. The reported O_2 demand of 2.05 kg_{O2} kg^{-1}_{CDW} also matches the respective stoichiometric demand. Pikaar et al. (2018)[8] considered electricity requirement for mixing and pumping of stirred fermentation reactors, as well as the energy demand for dewatering and drying of the broth from the fermentation step at 100 USD MWh^{-1}. The study considered CO_2 supply from a point source and three scenarios for the cost of H_2 supply (5, 3, and 0.7 USD kg^{-1}) by water electrolysers, leading to a total cost of 1460−3845 USD t^{-1}_{CDW}. The study concluded that H_2 supply is the main contributor to the total cost, followed by annuity of capital cost of SCP core plant, while the cost of process electricity and CO_2 and NH_3 supply would be marginal.

Nappa et al. (2020)[7] studied the production cost of 10,000 t_{CDW} $year^{-1}$ e-SCP production from electrolytic H_2 and point source CO_2 in Finland and Morocco. The study estimates a capex of about 11,1000 € t^{-1}_{CDW} a for the fermentation and downstream processing based on the installed equipment cost and 60% additional costs. However, no references are provided. Two scenarios are considered for electricity supply at each location. In the first scenario, PV with a capex of 999 € kW^{-1}, is the only source of power without consideration of balancing technologies. As such, water electrolyser and SCP core plant are forced to operate whenever PV electricity is available, which leads to over installation of electrolyser and SCP plant to maintain a certain annual SCP production. As a result, a SCP production cost of 24,000 and 10,700 € t^{-1}_{CDW} are reported for Finland and Morocco, respectively, for 10% rate of return. In the second scenario, grid electricity is used as a complementary source of power whenever PV electricity is not directly available. This leads to full-time operation of water electrolyser and SCP core plant, which in return lowers their installed capacity and impact of capital cost on the final product. Under this scenario, SCP production costs of 5300 and 5600 € t^{-1}_{CDW} are reported for Finland and Morocco, respectively. However, this scenario forces the use of potentially more expensive grid electricity for hydrogen production. The study considered a best scenario with an increased volumetric productivity of fermentation from 0.13 to 0.28 g_{CDW} L^{-1} h^{-1}, extended project technical lifetime from 20 to 30 years, and lowered grid electricity cost from 100 to 25 € MWh^{-1} in Morocco, leading to a SCP production cost of 2100 € t^{-1}_{CDW}. However, as the authors point out, none of these scenarios is ideal, as H_2 could be generated at the times low-cost electricity is available and balanced by relatively low-cost H_2 storage for a steady H_2 supply to the fermentation tanks. In addition, the study applies a capex of 1500 € kW^{-1} for PEM electrolysers in reference to the provided capex range by IEA. While such capex value is justified for medium-scale PEM electrolysers, we believe that cheaper and scalable alkaline electrolysers are better suited for large-scale projects.

García Martínez et al. (2021)[5] primarily studied the cost of e-SCP production for global protein supply in catastrophic conditions, such as such as nuclear winter, supervolcanic eruptions, or asteroid impacts. In this scenario, a 46% higher capital cost compared to normal conditions is considered for non-stop construction condition and a financial lifetime of 6 years. The authors

also investigated the production cost of e-SCP under normal condition and a financial lifetime of 20 years, which is further discussed here. The capital cost of SCP core plant is based on industrial estimations by Unibio A/S and NovoNutrients for a 108,000 t a^{-1} methane-based SCP plant at 251 mUSD[20]. The authors argue that this would be a conservative assumption due to slower growth rate of methane-oxidizing bacteria. The study considered a low-cost scenario wherein a grid electricity cost of 30 USD MWh^{-1}, an electrolyser capex of 440 USD kW^{-1}, free point source CO_2, and 80% protein content are considered. In the high-cost scenario, an electricity cost of 130 USD MWh^{-1}, an electrolyser capex of 888 USD kW^{-1}, atmospheric CO_2 supply by DAC, and 50% protein content are considered. Both scenarios are based on baseload grid electricity supply for all components, and a total electricity consumption of 8.2 MWh t^{-1}_{CDW} in the core SCP plant for fermentation, dewatering and drying steps. As such, the study reports on a respective minimum selling price of 4040−12,140 USD t^{-1}_{CDW} for a net present value range of zero. The financial assumptions include 70% equity financing with a 10% return on investment and the remaining 30% financed by a loan at 8% interest with a 10-year repayment term, as well as 35% revenue tax. Based on these assumptions, and the fact that the loan repayment time is half of the project lifetime, we calculated an average WACC of 9.28% to be used in cost recalculation based on the levelised cost method.

Leger et al. (2021)[14] performed a techno-economic assessment of e-SCP production by use of high-temperature aqueous solution CO_2 DAC and water electrolyser for H_2 and O_2 supply in a baseload system. The study considers a best-worst scenario approach based on 0.43−0.47 kg_{H2} kg^{-1}_{CDW}, 2.5−4 kg $t^{-1}_{H2,}$ 94−232 USD t^{-1}_{CO2}, 450−718 USD t^{-1}_{NH3}, 50−100 USD MWh^{-1} baseload electricity supply, 75−55% protein content, 2000−2300 USD t^{-1}_{CDW} a SCP core plant capex, 3−8% WACC, and 20 years lifetime. In addition, the total process energy requirement of SCP core plant is reported at 5.8−8.7 and 6.4−12 MWh t^{-1}_{CDW} for SCP for SCP as feed and food, respectively. Accordingly, e-SCP production costs of 1900−3400 and 1900−3800 USD t^{-1}_{CDW} are respectively reported for feed and food consumptions. The study also reports on an annual food protein production of 15 tonne per hectare, equivalent to a land usage of 667 m^2 per $t_{protein}$ a^{-1}, for PV-based systems in sunny regions with 2000 kWh m^{-2} annual irradiance and 75% protein content of the CDW. In our study, comparable regions have a land usage of 400−450 m^2 per $t_{protein}$ a^{-1} in 2030.

Jean and Brown (2024)[6] studied techno-economics of a 45,000 t a^{-1} SCP plant based on 11.1 USD MWh^{-1} baseload wind electricity, 988 USD kW^{-1} capex of water electrolyser for H_2 and O_2 supply, free CO_2 from corn ethanol plant, and point source NH_3 supply. The capital cost of SCP core plant is based on the earlier discussed industrial estimations by Unibio A/S and NovoNutrients for a 108,000 t a^{-1} methane-based SCP plants adjusted with a 0.6 scaling factor and consideration of 15% working capital, leading to an overall capex of 4600 USD t^{-1}_{CDW} a. Consequently, minimum selling price of 2070 USD t^{-1}_{CDW} is reported for a net present value of zero based on 50% loan at 6% interest and 20 years of project lifetime. Based on these assumptions, we calculated a WACC of 14.45% for the levelised cost approach. The study also considers a ±20% deviation from reference values in a series of sensitivity analyses. The reported results show that, at 144 USD t^{-1}_{CDW}, a 20% change in the capital cost of the SCP core plant has the highest impact on SCP production cost, followed by capex of electrolyser, cost of electricity and maintenance at 87, 49 and 45 USD t^{-1}_{CDW}, respectively. In this study, the average wholesale market price of wind in the Midcontinent Independent System Operator (MISO) region of USA in 2020, at 11.1 USD MWh^{-1}, is considered as the cost of baseload electricity supply to the system. In our view, such assumption may underestimate the cost of baseload electricity supply, as average price of wind electricity represents the price at the time of wind electricity generation, which is not comparable to the price of baseload electricity supply that would impose additional costs for electricity balancing technologies. In addition, the study considers electricity consumption by other processes negligible compared to electrolyser. However, as shown in our study and earlier studies[5,7,14], the total additional electricity demand in the system is significant and could be up to 70% of the electricity demand by electrolysers. Such underestimation of the total electricity demand will further underestimate the impact of electricity cost on SCP production cost. Jean and Brown (2024)[6] also report on an annual protein production of 276 tonne per hectare, equivalent to a land usage of 36 m^2 per $t_{protein}$ a^{-1}, for a wind-based power system and 65% protein content of the CDW. However, we found such land use rate at least an order of magnitude too low.

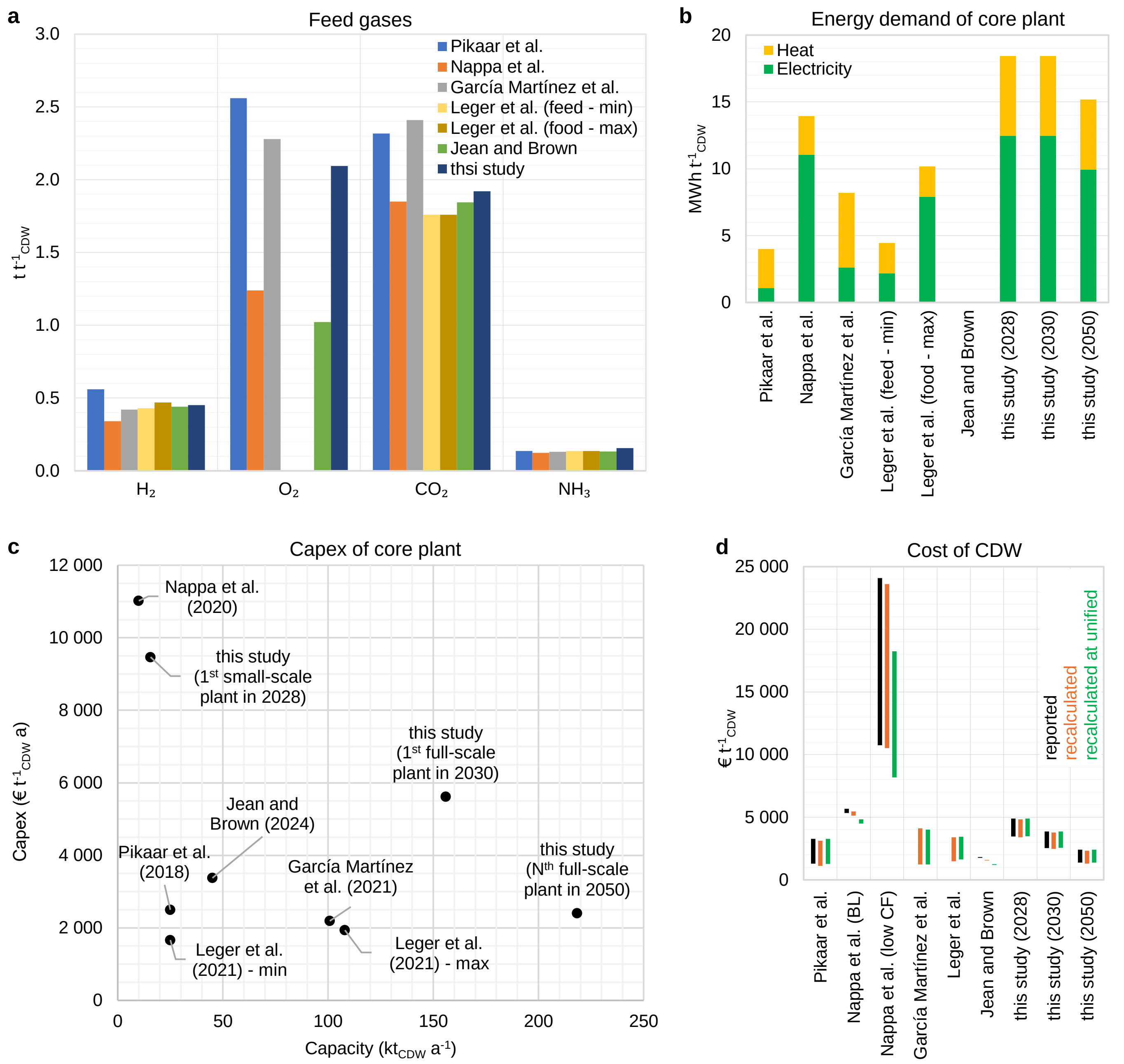

Supplementary Figure 2 | Comparision of this study with literature. a Feed gases. **b** Energy demand of core plant. **c** Capex of core plant. **d** Cost of CDW. It should be noted that Leger et al.[14] calculated low and high H_2 demand values of 0.43 and 0.47 kg kg^{-1}_{CDW}, respectively, in Dataset_S01, tab 'SCP Cost Estimation (S1G)', cells AK50:AL50. However, the values were reversed in the H_2 cost calculations in cells M7:N7. In this study, we used the correct order for H_2 demand and recalculated CDW cost production costs, while retaining the original reported CDW costs. This adjustment resulted in higher and lower reproduced CDW costs compared to the originally reported values for the low and high scenarios, respectively. Abbreviation: cell dry weight (CDW). Source data are provided as a Source Data file.

Supplementary Note 4. Long-term development and techno-economics data on Solar Foods SCP core plant

Supplementary Table 5. Development of SCP plants installation and costs, based on SCP content. Abbreviation: learning rate (LR).

	Unit	2028	2030	2035	2040	2045	2050	2055	2060	2065	2070
Reference Scenario											
operational capacity	Mt_{SCP} a^{-1}	0.01642	0.181	1.66	6	18	48	93	127.5	142.5	150
historical cumulative capacity	Mt_{SCP} a^{-1}	0.0164	0.181	1.66	6	18	48	93.2	129.4	150.4	175.9
doublings between periods	[-]	–	3.46	3.2	1.86	1.58	1.42	0.96	0.47	0.22	0.23
capex - 10% LR	€ $kg^{-1}{}_{SCP}$ h	71,960	42,728	30,504	25,088	21,232	18,288	16,536	15,736	15,384	15,024
opex fixed											
maintenance & insurance	% of capex	4	4	4	4	4	4	4	4	4	4
personnel - 5% LR	€ $t^{-1}{}_{SCP}$ a	113	33.9	28.8	26.1	24.1	22.4	21.3	20.8	20.6	20.4
others - 5% LR	€ $t^{-1}{}_{SCP}$ a	30.6	13.3	11.3	10.2	9.4	8.8	8.4	8.2	8.1	8
$opex_{var}$	€ $t^{-1}{}_{SCP}$	75	75	73	70	69	67	67	67	67	67
Advanced Scenario											
operational capacity	Mt_{SCP} a^{-1}	0.01642	0.181	3.32	12	36	96	186	255	285	300
historical cumulative capacity	Mt_{SCP} a^{-1}	0.016	0.181	3.32	12	36	96	186	259	301	352
doublings between periods	[-]	–	3.46	4.2	1.86	1.58	1.42	0.96	0.47	0.22	0.23
capex - 15% LR	€ $kg^{-1}{}_{SCP}$ h	71,960	42,728	21,592	15,968	12,344	9808	8400	7776	7504	7232
opex fixed											
maintenance & insurance	% of capex	4	4	4	4	4	4	4	4	4	4
personnel – 7.5% LR	€ $t^{-1}{}_{SCP}$ a	113	33.9	24.4	21.1	18.7	16.7	15.5	15	14.7	14.5
others – 7.5% LR	€ $t^{-1}{}_{SCP}$ a	30.6	13.3	9.6	8.3	7.3	6.6	6.1	5.9	5.8	5.7
$opex_{var}$	€ $t^{-1}{}_{SCP}$	75	75	73	70	69	67	67	67	67	67

Abbreviation: learning rate (LR).

Supplementary Table 6. Development of SCP plants installation and costs, based on protein content.

	Unit	2028	2030	2035	2040	2045	2050	2055	2060	2065	2070
operational capacity - Ref. Scenario	$Mt_{protein}$ a^{-1}	0.0101	0.112	1.02	3.71	11.1	29.6	57.4	78.7	88.0	92.6
operational capacity - Adv. Scenario	$Mt_{protein}$ a^{-1}	0.0101	0.112	2.05	7.41	22.2	59.3	114.9	157.5	176.0	185.3
historical cumulative capacity - Ref. Scenario	$Mt_{protein}$ a^{-1}	0.0101	0.112	1.02	3.71	11.1	29.7	57.5	79.9	92.8	108.6
historical cumulative capacity - Adv. Scenario	$Mt_{protein}$ a^{-1}	0.0101	0.112	2.05	7.41	22.2	59.3	115.0	159.6	185.6	217.1
installed capacity - Ref. Scenario	$Mt_{protein}$ $timestep^{-1}$	0.0101	0.101	0.91	2.68	7.4	18.5	27.9	22.3	13.0	15.7
installed capacity - Adv. Scenario	$Mt_{protein}$ $timestep^{-1}$	0.0101	0.101	1.83	5.36	14.8	37.1	55.8	44.7	25.9	31.5
capex - Ref. Scenario	€ $t^{-1}{}_{protein}$ a	14567	8649	6175	5079	4298	3702	3347	3185	3114	3041
capex - Adv. Scenario	€ $t^{-1}{}_{protein}$ a	14567	8649	4371	3232	2499	1985	1700	1574	1519	1464

Abbreviations: reference (Ref.), advanced (Adv.).

5.5. This study

The cost of a cluster of alkaline water electrolysers in 2020 is provided in Supplementary Table 7. The total cost and efficiency of the 28 MW electrolyser system at 5 bar is based on a quote from a European original equipment manufacturer (OEM), with additional costs on the buyer's side evaluated by an engineering consulting company. Due to modularity of electrolyser units, the impact of economies of scale diminishes for larger systems. The cost of the 50 and 250 MW electrolyser systems at 5 bar are based on a scaling factor of 0.93 for expansion from 28 to 50 MW, and a scaling factor of 0.95 for expansion from 50 to 250 MW. We consider an output pressure of 30 bar common for future electrolysers. Thus, for hydrogen delivery at 30 bar, additional cost of H_2 compressor units at similar hydrogen throughput as the respective electrolyser system are included. The additional power consumption by the H_2 compressor is not included in the overall efficiency, as standard high-pressure alkaline electrolysers already have similar or higher system efficiencies[21].

Supplementary Table 7. Cost of a cluster of alkaline water electrolysers in 2020.

Electrolyser capacity	MW	28	50	250	reference
	$MW_{H2,HHV}$	20.5	36.7	183.3	
stack and balance of stack (5 bar)	M€	11.83			a 2019 quote from a European OEM
additional services by supplier [a]	M€	2.32			a 2019 quote from a European OEM
additional costs by buyer [b]	M€	3.71			SWECO Finland
installed system (5 bar) - capital cost	M€	17.86	30.62	141.28	50 and 250 MW systems based on scaling factor
installed system (5 bar) - capex	€ kW^{-1}	638	612	565	
H_2 compressor capacity	MW	0.57	1.02	5.08	based on 28 kWh_{el} $MWh^{-1}_{H2,HHV}$ from [22]
H_2 compressor cost	M€	2.64	3.43	8.66	based on [23]
installed system (30 bar) - capital cost	M€	20.5	34.05	149.94	
installed system (30 bar) - capex	€ kW^{-1}	732	681	600	
	€ $kW^{-1}_{H2,HHV}$	999	929	818	

Abbreviation: original equipment manufacturer (OEM).
[a] freight, installation, commissioning, and training.
[b] project management, engineering, permits, civil work, building, HVAC, foundation, utilities, and spare parts.

The projected capex development of 250 MW alkaline water electrolyser systems by 2050 is provided in Supplementary Table 8. It is based on the impact of the learning rate[24–27] for increasing the total operational capacity of water and chlor-alkali electrolysers from 30 GW in 2020 to ~10 TW by mid-century in an S-curve development pattern. The projected increase in the operational capacity of electrolysers is mostly related to water electrolyser for supply of 50% of required hydrogen for e-fuels[28] and e-chemicals[29] in a 100% renewable energy system by 2050.

For comparison, the projected total chlor-alkali and water electrolyser operational capacity of 499 GW by 2030 is less than the 600 GW water electrolyser capacity required by 2030 in the IEA scenario for net zero emissions by 2050[30]. In terms of achievability, our target capacity is in line with the 420 GW of water electrolyser announced projects (final investment decision, feasibility, or early stage) by 2030, according to the IEA[30]. In the coming years, more projects could be expected to be announced for this timeframe.

Chlor-alkali electrolysers are included due to their similarities to water electrolysers and the spillover effect on the learning rate. The inclusion of chlor-alkali electrolysers increases the cumulative installed capacity of electrolysers in 2020 from ~14 GW to 52 GW. Accordingly, the number of doublings of cumulative installed capacity and consequently the impact of the learning rate on capex reduction by new installations declines. It is likely that the operational capacity and cumulative installed capacity of electrolysers in 2020 are overestimated in this study, which leads to even smaller impact of learning rate on capex reduction by new installations. Moreover, we consider a declining learning rate over time (Supplementary Table 8).

Supplementary Table 8. Capex development of alkaline water electrolyser (250 MW system at 30 bar).

	Unit	2020	2025	2030	2035	2040	2045	2050
Water and chlor-alkali electrolyser								
operational capacity	GW_{el}	30	124	499	1558	3658	6668	9980
newly installed capacity	GW_{el}	6.6	93	375	1060	2099	3011	3319
cumulative installed capacity	GW_{el}	52	145	520	1580	3679	6689	10,008
Water electrolyser - 250 MW_p at 30 bar								
learning rate	%	18	18	17	17	16	16	15
capex	€ kW^{-1}_{el}	600	446	316	234	189	163	148

5.2. Comparison with the literature

The cost of electrolyser plants at capacities below 10 MW is sharply affected by economies of scale[31,32]. Thus, for comparison to other references, electrolysers of comparable size should be considered. A cost comparison with recent and log-term large-scale electrolyser systems is provided in Supplementary Table 9. The table includes a list of recent publicly announced large-scale purchase orders (PO) from NEL Hydrogen[33], academic literature, as well as short-term and long-term capex estimations by the IEA and BloombergNEF.

The NEL's POs mainly report on the stack cost and do not present the full cost of the electrolyser system. Nevertheless, they provide an up-to-date industry insight on the cost of main component, as well as the impact of time and scale. NEL aims to reduce the cost of stack by 70% from 2022 to 2026[34], equivalent to a compound annual reduction of 26%. In addition, NEL estimates the cost of balance of stack (BoS) at the same level as the cost of the stack[34]. According to the data by OEM quote and SWECO Finland in Supplementary Table 7, the additional costs of electrolyser plant full installation are about 50% of the cost of stack and balance of stack. Thus, the full installation cost for a 28 MW in 2020 could be estimated at about 3 times the stack cost. As a rough estimation, assuming the same ratio holds for larger projects in future, the reported PO for 200 MW stack in 2023 at 225 € kW^{-1} is equivalent to a system-level capex of about 675 € kW^{-1}. This capex is about 19% higher than our 2020 capex of 565 € kW^{-1} for a 250 MW system at 5 bar. The capex of 40 MW stack systems to be delivered in 2025 is about 275−300 € kW^{-1}, equivalent to 825−900 € kW^{-1} for the overall system. For comparison, we project a capex of 612 € kW^{-1} for a 50 MW electrolyser system at 5 bar in 2025. However, the European economy experienced significant inflation in the early 2020s due to the global pandemic and the war in Ukraine. For a better comparison, the capex values are compared for a 250 MW system in 2019 € value based on Chemical Engineering Plant Cost Index (CEPI: 2019 (607.5), 2020 (596.2), 2021 (708.8), 2022 (816.0), 2023 (797.9)). As such, the capex values become mostly at the same ballpark as our chosen capex for 2020.

Chinese electrolysers, on the other hand, reportedly have lower costs. According to electrolyser industry insights[35] as of end of 2023, the price of electrolysers in China is about 200 USD kW^{-1} (~167 € kW^{-1}). Such price level is reported to have been achieved by several technology providers, including the two largest solar PV manufacturers and a fully owned subsidiary of the largest European manufacturer. However, such cost does not include balance of plant, and it is not clear if it includes balance of stack either. In addition, these electrolysers have reportedly under delivered and faced challenges with safety, efficiency and flexibility. The electrolysers from prominent Chinese manufacturers have had only 30% availability in Sinopec's 260 MW green hydrogen project in China, due to the choice of material. According to BloombergNEF, these problems are expected to be resolved in the next 5 years as the industry matures. However, it is yet to be seen whether overcoming such challenges would lead to an increase in the cost of Chinese electrolysers.

According to a BloombergNEF's 2024 survey[36], in the past two years, the total system cost of electrolysers has increased to 480-720 USD kW^{-1} (400-600 € kW^{-1}) in China and 2000-3000 kW (1667-2500 € kW^{-1}) in Europe and the US, due to inflation and larger scope of cost coverage. In addition, the installed system cost of Chinese electrolysers in Europe could be expected to be higher than their installed system cost in China, due to shipping and higher labour and installation costs. Assuming 100 € kW^{-1} additional cost for shipping and installation of the electrolysers in Western countries

would increase the total cost of Chinese electrolysers installed in Europe to 500 € kW^{-1} for large-scale projects in 2023, equivalent to 381 €$_{2019}$ kW^{-1}. Respectively, the 2000 € kW^{-1} capex for large-scale projects in Europe is equivalent to about 1500 €$_{2019}$ kW^{-1}. Such cost differences may lead to more market consolidation towards the lower range of the cost level, as projected in a 2019 report by BloombergNEF[29].

A 2023 report by IEA[30] also acknowledges the lower cost of Chinese electrolysers and a general cost increase in the early 2020s. According to this report, the installed cost of alkaline electrolysers increased by 15% in 2021-2023 to 1700 USD kW^{-1} due to inflation and labour cost. However, they conclude that the costs could be reduced to ~850 and 600−700 USD kW^{-1} in 2025 and 2030, respectively. According to this IEA report, the cost of Chinese electrolyser in 2023 was 350−1300 USD kW^{-1}, higher than the cost estimation by BNEF, yet significantly lower than the electrolyser cost in Europe[30].

Our 2020 capex of 600 €$_{2019}$ kW^{-1} for 250 MW electrolyser systems in 2020 is within the capex range provided by BloombergNEF. Nevertheless, as a sensitivity analysis, an 800 €$_{2019}$ kW^{-1} for 250 MW electrolyser systems in 2020 is considered as the starting point capex development and e-protein production cost in 2030 and 2050.

In terms of levelised cost, in 2023, BloombergNEF projected that the cost of green hydrogen declines from 2.5−11 USD kg^{-1} (53−233 € MWh$^{-1}_{H2,HHV}$) in 2023, to 1−4 USD kg^{-1} (21−85 € MWh$^{-1}_{H2,HHV}$) in 2030, and below 2 USD kg^{-1} (42 € MWh$^{-1}_{H2,HHV}$) beyond 2035 at different countries around the world[37]. Our results in Fig. 6 shows an electricity generation cost of 20−23 € MWh^{-1} and an electrolyser FLh of 3000−3300 hours at best solar sites in 2030. As such, based on the specifications of water electrolyser in this study, the cost of hydrogen generation in 2030 would be 1.9−2.2 USD kg^{-1}. While such hydrogen generation costs are within the projected range by BloombergNEF in 2030, it could be argued that the results of this study are relatively conservative as they represent some of the least cost locations in the world that does not come close to the lower limit projected by BloombergNEF.

Supplementary Table 9. Literature review on capex of large-scale alkaline electrolyser.

Year of PO or report	Year	Capacity	Total capex	Unit capex	Estimated system capex	Estimated capex at 250 MW	Estimated capex at 250 MW	Location	Scope/Comment	ref.
		MW	M€	€ kW^{-1}	€ kW^{-1}	€ kW^{-1}	€$_{2019}$ kW^{-1}			
2019.05	2020	28	17.86	638	638	565	565		installed system cost – 5 bar	European OEM
2020.12	2021	20	7.2	360	1080	935	953	Denmark	stack	NEL PO
2021.11	2022	20	11	550	825	714	612	Sweden	stack and BoS - greenfield	NEL PO
2022.07	2023	200	45	225	675	668	497	USA	stack	NEL PO
2023.07	2023	20	9	450	675	584	445	France	stack and BoS (?)	NEL PO
2022.11	2023	40	12	300	900	818	609	Norway	stack	NEL PO
2024.03	2023	N.A.			1667	1667	1269	Europe & US	installed system cost	BNEF (2024)[36]
2024.03	2023	N.A.			400	400	305	China	installed system cost	BNEF (2024)[36]
2024.03	2023	N.A.			500	500	381	Europe & US	Chinese electrolyser + 100 € kW^{-1} higher installation	based on BNEF 2024
2023	2023	N.A.		1700	1700	1700	1294		installed system	IEA (2023)[30]
2023	2023	N.A.		300−1100	300−1100	300−1100	228-838		installed system - China	IEA (2023)[30]
2023.12	2024	N.A.			167	167	127	China		H_2 Insight (2023)[35]
2023.07	2025	40	11	275	825	749	570	Portugal	stack	NEL PO
2023.02	2025	40	12	300	900	818	623	Netherlands	stack (?)	NEL PO
2023	2025	N.A.		700	700	700	533		installed system	IEA (2023)[30]
Long-term										
2023	2030			372−564	372−564	372−564	283-429		bottom-up results	Krishnan et al. [38]
2023	2030			524−1166	524−1166	524−1166	399-888		learning rate	Krishnan et al.[38]
2023	2030			972	972	972	740			Glenk and Reichelstein[39]
2023	2030	N.A.		500-580	500-580	500-580	381-442		installed system	IEA (2023)[30]
2020	2050	N.A.			130-200		130-200		installed system	IRENA (2020)[40]

Abbreviations: purchase order (PO), original equipment manufacturer (OEM), and balance of plant (BoP).

Supplementary Note 6. Energy and cost projection of solid sorbent Direct Air Capture

The current and projected electrical and thermal energy demand of low temperature solid sorbent DAC technologies varies significantly in the literature, as shown in Supplementary Table 10. The list includes data published over time by Climeworks (the only company with commercial scale solid sorbent DAC plants), recent academic literature, and a potential range of energetic demands by the National Academies of Sciences, Engineering, and Medicine.

Supplementary Table 10. Energy demand of solid sorbent direct air capture plants in the literature.

Reference	Electricity demand	Heat demand	Comment
	kWh_{el} t^{-1}_{CO2}	kWh_{th} t^{-1}_{CO2}	
Climeworks factsheet (2018)[41]	700	2200	including electricity demand for carbon storage process
Beuttler et al. (2019)[42]	400	1600	long-term projections by Climeworks for DACCS
Climeworks website FAQ (2021)[43]	650	2000	accessible via https://archive.org/web/
Deutz and Bardow (2021)[44]	700	3300	based on Climeworks' Artic Fox unit in Iceland
	500	1500	future target values
Climeworks (2023)[45]	direct numbers not available		Climeworks' next generation technology is expected to halve the energy demand compared to the Orca plant
National Academies of Sciences, Engineering, and Medicine (2019)[46]	22	514	best scenario – a scenario that may be unachievable
	156	944	low scenario
	315	1333	high scenario
Sabatino et al. (2021)[47]	80–160	1000–2500	based on various sorbents and isotopes of CO_2 and water (excl. outliers)
Sendi et al. (2022)[48]	250	1930	CO_2 capture at 20 °C and 50% relative humidity and compression to 150 bar
	160	1930	excluding approximate electricity demand for CO_2 compression to 150 bar
Wiegner et al. (2022)[49]	329–347	1704–1820	CO_2 capture at 20 °C and 75% relative humidity (excluding outlying data)

Reliable data on investment cost of solid sorbent DAC plants based on actual plants is scarce. In this study, the current and near future energy demand and capex of low-temperature solid sorbent DAC are based on Climeworks' current and next generation plants (Supplementary Table 11). With 4000 t_{CO2} per year capacity, the Orca plant in Iceland is Climeworks' largest solid sorbent DAC plant operational since 2021. The capex of the Orca plant for carbon capture and sequestration is reported at 10–15 mUSD[50]. We consider an average of 12.5 mUSD for carbon capture only (excluding the sequestration process) with a long-term USD €$^{-1}$ exchange rate of 1.2. So far, the average energy consumption of the Orca plant has not been disclosed. However, it has been mentioned that the Orca plant has not been optimised for energy efficiency. Thus, we estimate its electricity demand to be same as earlier plants at 700 kWh t^{-1}_{CO2}, and its heat demand at 3000 kWh_{th} t^{-1}_{CO2}, considering some improvements compared to the heat demand of the Artic Fox DAC unit. Climeworks next project (Mammoth) is based on the same technology as the Orca plant and is expected to become operational in 2024. Regardless of 9 times larger capacity compared to Orca, Climeworks expects similar capex for the Mammoth plant. This is because addressing the problems identified during the operation of the Orca plant increases the capex of the Mammoth plant that offsets the benefit of the economy of scale. In the Direct Air Capture Summit 2023[45], Climeworks revealed that their next project after Mammoth would be based on their next generation technology and about 10 times larger. The next generation technology is reported to have two times higher output density, leading to a scaling factor of 0.7 for a 10 times larger plant compared to the Mammoth plant. The next generation technology is also reported to reduce the energy demand by 50% compared to the Orca and Mammoth plants[45]. Since Climeworks has not distinguished the electrical and thermal efficiency gains, we consider the same reduction rate for both. Nevertheless, both electrical and thermal energy demand remain within the feasible range in the literature.

Supplementary Table 11. Specifications of Climeworks' current and near future direct air capture plants.

Project name	Unit	Orca	Mammoth	Next generation technology	comment
Year		2021	2024	~2027	
Capacity	kt_{CO2} a^{-1}	4	36	360	
Full load hours	h	8000	8000	8000	self-assumption
Capital cost	M€	10.4	93.7	564	
Capex	€ t^{-1}_{CO2} a	2378	2378	1192	
	€ kg^{-1}_{CO2} h	20,833	20,833	10,441	
Electricity demand	kWh_{el} t_{CO2}	700	700	350	self-estimation
Low-temperature heat demand	kWh_{th} t_{CO2}	3000	3000	1500	self-estimation

According to the International Energy Agency (IEA), a total of 1.85 Mt_{CO2} a^{-1} of solid sorbent DAC projects are already at different stages for deployment by 2027[51]. We then consider a S-curve development of the operational capacity of solid sorbent DAC to 2.5 Gt_{CO2} a^{-1} by 2050 (Supplementary Table 12), well below estimations for required DAC capacity by mid-century[52]. The capex and energy demand of DAC in 2028−2050 are then calculated based on the cumulative installed capacity of DAC at each time-step and the respective learning rates. A capex learning rate of 10−18% and an energy consumption learning rate of up to 10% are often considered for modular low-temperature solid sorbent DAC plants[45,53]. In this study, a learning rate of 12% and 4% are considered for capex and energy demand of low-temperature DAC, respectively. As such, the projected electricity and heat demand of DAC plants in 2050 (Supplementary Table 12) remain well above the "low scenario" by the National Academies of Sciences, Engineering, and Medicine provided in the Supplementary Table 10. Young et al.[53] report on a sorbent cost or 37 USD t^{-1}_{CO2} and a learning rate of 10−18% learning rate. A learning rate of 10% for the sorbent is used in this study.

Supplementary Table 12. Projected long-term specifications of DAC plants.

	Unit	2020/21	2024/25	2027	2028	2030	2035	2040	2045	2050
Cumulative capacity	Mt_{CO2} a^{-1}		0.5	1.85	3.7	15	110	550	1375	2500
Unit capacity	kt_{CO2} a^{-1}	4	36	360	360	360	360	360	360	360
Capex	€ t^{-1}_{CO2} a	2378	2378	1192	1049 [a]	810	561	417	352	315
	€ kg^{-1}_{CO2} h	20,833	20,833	10,441	9189	7096	4914	3653	3084	2759
Electricity demand	kWh_{el} t^{-1}_{CO2}	700	700	350	336	309	275	250	237	229
Low-temperature heat demand	kWh_{th} t^{-1}_{CO2}	3000	3000	1500	1440	1326	1179	1072	1016	981
Sorbent cost	€ t^{-1}_{CO2}	31	31	25.4	22.9	18.5	13.7	10.7	9.3	8.5

[a] For the small-scale Power-to-SCP plant in 2028, a 10 times smaller DAC plant (36 kt_{CO2} a^{-1}) would be sufficient. Considering a scaling factor of 0.9 for modular DAC plants, the capex of the small-scale DAC plant increases by 26% to 1321 € t^{-1}_{CO2} a in 2028.

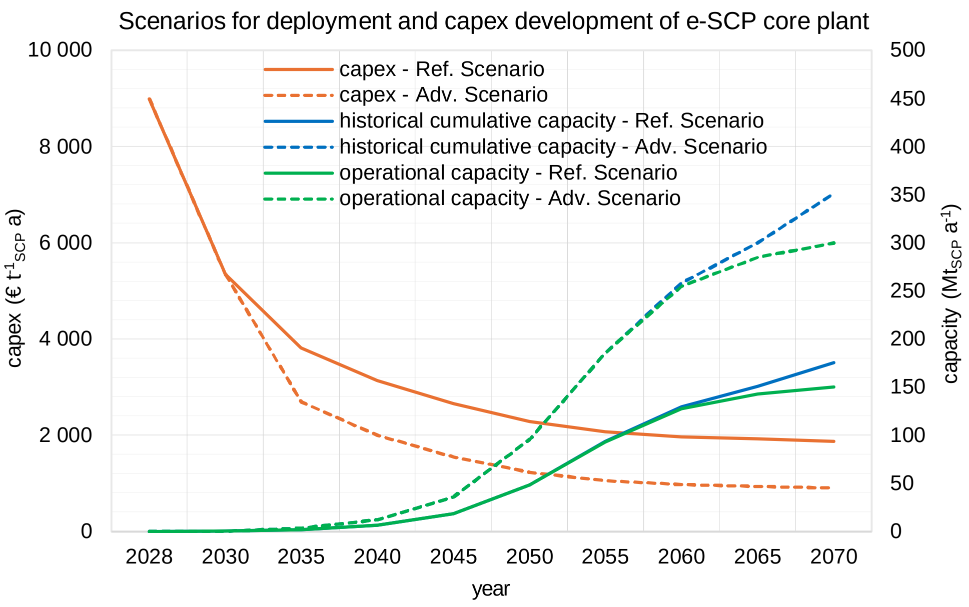

Supplementary Figure 3 │ Potential capex decline of e-SCP core plant based on deployment, learning rate, and scenarios. Source data are provided as a Source Data file.

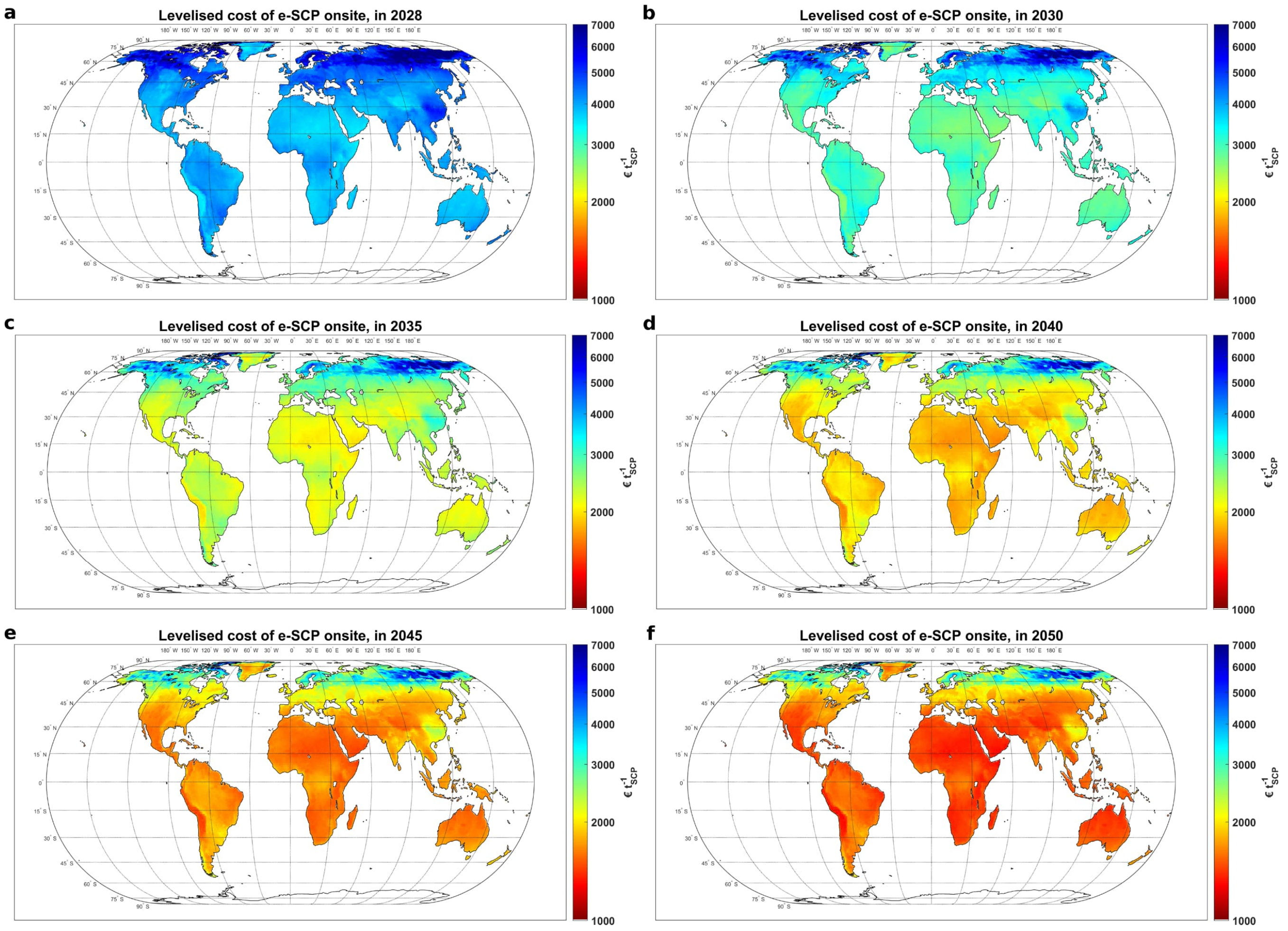

Supplementary Figure 4 │ Levelised cost of RE-based Single-Cell Protein from 2028 to 2050. a in 2028. **b** in 2030. **c** in 2035. **d** in 2040. **e** in 2045. **f** in 2050. Source data are provided as a Source Data file.

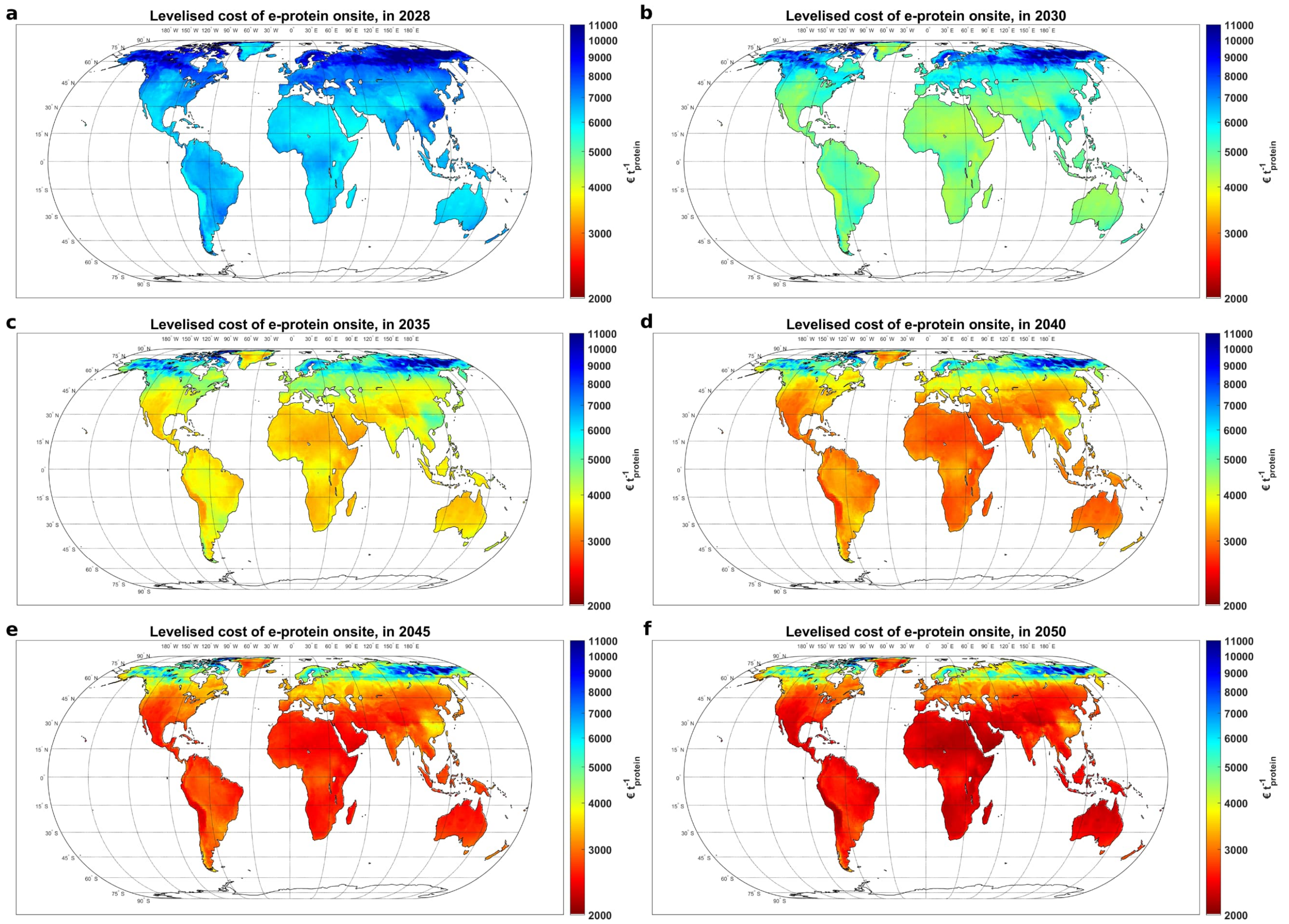

Supplementary Figure 5 | Levelised cost of e-protein from 2028 to 2050. a in 2028**. b** in 2030. **c** in 2035. **d** in 2040. **e** in 2045. **f** in 2050. Source data are provided as a Source Data file.

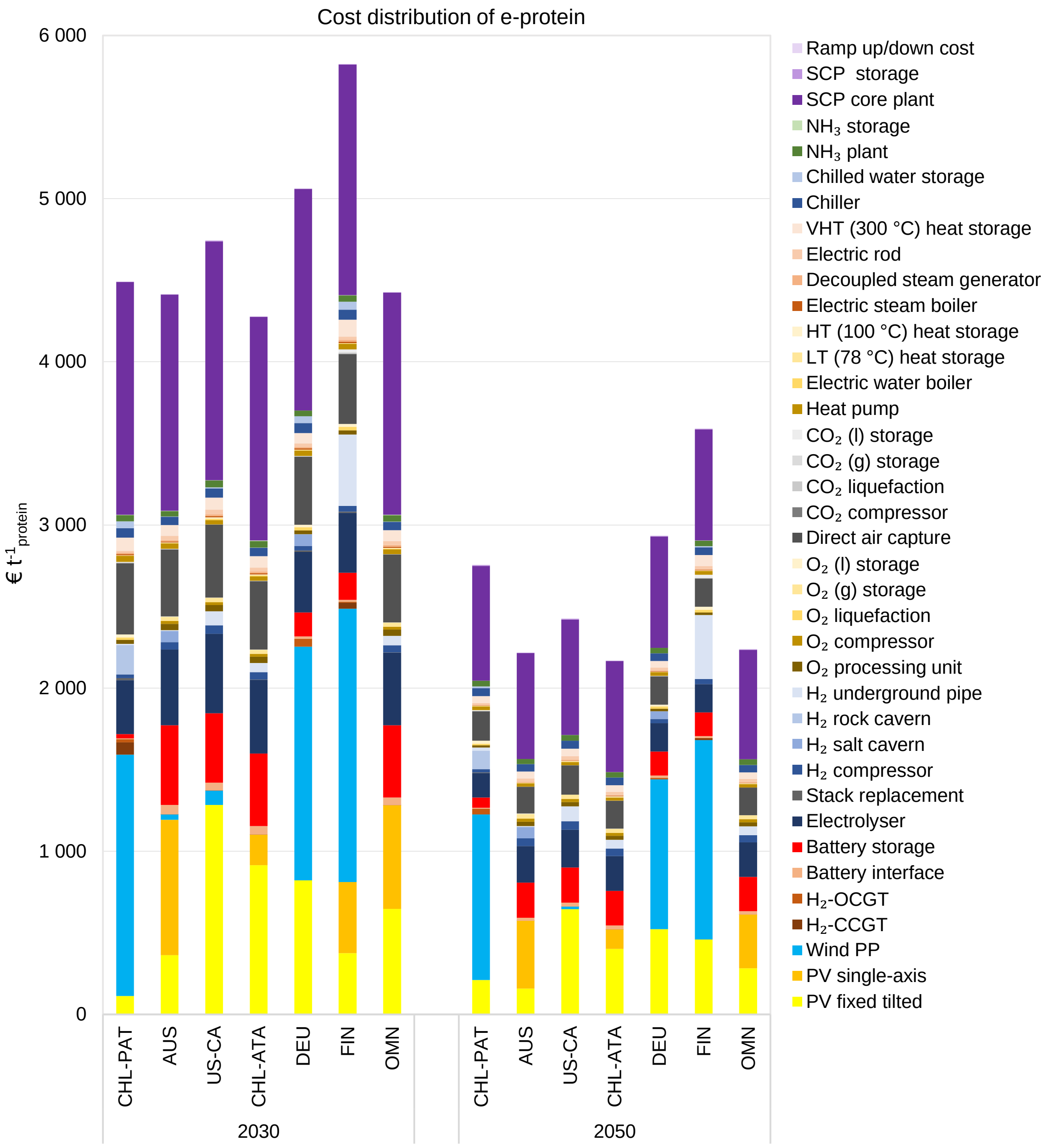

Supplementary Figure 6│ Cost distribution of e-protein by components in 7 nominated sites in 2030 and 2050. Abbreviations: open cycle gas turbine (OCGT), closed cycle gas turbine (CCGT), low temperature (LT), high temperature (HT), and very high temperature (VHT). Source data are provided as a Source Data file.

Supplementary Table 13. Location of the seven nominated sites for e-SCP plants.

Abbreviation	CHL-PAT	AUS	US-CA	CHL-ATA	DEU	FIN	OMN
Location	Chile - Patagonia	Australia	US - California	Chile - Atacama	Germany	Finland	Oman
[Lat, Lon]	[-52.65, -72.45]	[-18.9, 123.75]	[35.1, -117]	[-23.85, -69.3]	[54, 12.6]	[63.45, 22.5]	[22.5, 58.95]

Supplementary Table 14. Electricity consumption by sub-units for e-protein supply via hybrid PV-wind Power-to-SCP supply chain in 2030.

Item	Unit	CHL-PAT	AUS	US-CA	CHL-ATA	DEU	FIN	OMN
Electrolyser	kWh $t^{-1}_{protein}$	40,910	38,091	38,081	38,089	39,549	38,935	38,081
H_2 compressor	kWh $t^{-1}_{protein}$	242	399	399	407	265	258	408
O_2 compressor	kWh $t^{-1}_{protein}$	0	62	64	67	0	0	68
O_2 liquefaction	kWh $t^{-1}_{protein}$	170	23	19	9	208	223	4
Direct air capture	kWh $t^{-1}_{protein}$	1031	1031	1031	1031	1031	1031	1031
CO_2 compressor	kWh $t^{-1}_{protein}$	4	1	1	0	1	1	0
CO_2 liquefaction	kWh $t^{-1}_{protein}$	6	4	1	0	3	17	1
Heat pump	kWh $t^{-1}_{protein}$	1109	1089	730	851	1116	1117	994
Electric water boiler	kWh $t^{-1}_{protein}$	35	109	1386	954	10	9	446
Electric steam boiler	kWh $t^{-1}_{protein}$	1743	1144	675	969	1564	1190	1101
Electric rod	kWh $t^{-1}_{protein}$	8071	8676	9147	8851	8222	8665	8713
Chiller	kWh $t^{-1}_{protein}$	5461	5461	5461	5461	5461	5461	5461
NH_3 plant	kWh $t^{-1}_{protein}$	177	177	177	177	177	177	177
SCP core plant	kWh $t^{-1}_{protein}$	13,725	13,725	13,725	13,725	13,725	13,725	13,725
Battery loss	kWh $t^{-1}_{protein}$	24	972	780	882	172	172	869
Curtailed electricity	kWh $t^{-1}_{protein}$	1839	2410	5296	3591	2557	7928	4161

Supplementary Table 15. Installed capacities of Power-to-SCP subunits for 100 kt a^{-1} e-protein supply in 2030.

Item	Unit	CHL-PAT	AUS	US-CA	CHL-ATA	DEU	FIN	OMN
PV fixed tilted	MW	378.8	1215.2	4297.8	3061.6	2751.5	1255.4	2167.6
PV single-axis	MW	0.0	2524.0	0.0	568.0	0.0	1328.3	1932.5
Wind power plant	MW	1398.4	31.3	84.0	0.0	1354.1	1582.8	0.0
H_2-CCGT	MW	74.0	0.1	0.0	0.0	0.1	35.9	0.0
H_2-OCGT	MW	30.7	0.6	0.0	0.7	76.5	9.4	0.0
Battery interface	MW	70.6	902.5	770.1	836.1	227.7	189.4	741.9
Battery storage	MWh	219.6	3869.8	3 357.2	3510.1	1171.3	1328.2	3495.6
Electrolyser	$MW_{H2,HHV}$	615.9	900.3	949.3	877.8	702.0	695.9	861.0
H_2 compressor	$MW_{H2,HHV}$	312.0	613.6	716.5	619.0	391.1	472.6	596.4
H_2 salt cavern	$MW_{H2,HHV}$	0.0	124,468.2	0.0	0.0	131,816.9	0.0	0.0
H_2 rock cavern	$MW_{H2,HHV}$	111,582.4	306.1	0.0	0.0	0.0	0.0	0.0
H_2 underground pipe	$MW_{H2,HHV}$	738.7	412.1	8002.5	5143.5	1.3	41,485.6	5367.1
O_2 processing unit	t_{O2} h^{-1}	55.8	92.3	93.5	92.4	57.3	61.6	91.0
O_2 compressor	t_{O2} h^{-1}	0.0	56.8	58.1	59.1	0.1	0.2	59.3
O_2 liquefaction	t_{O2} h^{-1}	16.1	3.2	2.6	1.4	20.2	23.1	0.6
O_2 (g) storage	t_{O2}	0.4	480.7	501.3	507.6	0.6	2.4	524.3
O_2 (l) storage	t_{O2}	4151.3	763.0	288.2	175.1	3317.7	3809.7	72.8
Direct air capture	t_{CO2} h^{-1}	41.1	38.3	42.3	39.5	39.0	40.4	39.1
CO_2 compressor	t_{CO2} h^{-1}	3.8	0.6	0.4	0.1	1.8	0.5	0.2
CO_2 liquefaction	t_{CO2} h^{-1}	1.5	0.4	0.2	0.1	0.8	5.1	0.2
CO_2 (g) storage	t_{CO2}	150.1	23.8	16.0	2.2	55.0	23.1	4.8
CO_2 (l) storage	t_{CO2}	281.2	314.0	58.8	10.9	147.0	2 737.8	51.5
Heat pump	MW_{th}	50.2	46.1	38.0	39.6	48.1	49.8	42.8
Electric water boiler	MW_{th}	0.7	5.5	41.6	24.0	0.7	0.3	14.8
LT (78 °C) heat storage	MWh_{th}	264.2	271.5	193.7	233.9	262.3	288.3	251.2
HT (100 °C) heat storage	MWh_{th}	7.1	35.4	224.3	125.8	7.7	4.9	83.7
Electric steam boiler	MW_{th}	73.2	62.1	77.2	84.3	98.5	101.9	77.1
Decoupled steam generator	MW_{th}	115.0	109.9	121.8	113.6	112.8	117.7	113.1
Electric rod	MW_{th}	134.3	302.6	364.1	310.6	256.1	259.0	292.8
VHT (300 °C) heat storage	MWh_{th}	2218.3	1837.5	2015.4	1874.3	1729.1	2795.0	1831.1
Chiller	t_{ChW} h^{-1}	40,635.1	35,379.4	39,988.1	36,514.0	43,180.9	43,710.4	36,267.0
Chilled water storage	t_W	319,745.4	17,633.9	52,048.0	12,219.6	320,330.0	368,563.0	10,126.8
NH_3 plant	t_{NH3} h^{-1}	3.8	3.3	4.1	3.9	3.3	3.9	3.9
NH_3 storage	t_{NH3}	1435.7	796.6	1500.8	2731.6	548.7	1184.1	1651.7
SCP core plant	t_{SCP} h^{-1}	22.6	20.8	23.2	21.6	21.4	22.4	21.5
SCP storage	t_{SCP}	6265.5	2789.5	10,997.8	4928.8	3591.9	6996.2	4509.6

Abbreviations: open cycle gas turbine (OCGT), closed cycle gas turbine (CCGT), low temperature (LT), and high temperature (HT).

Supplementary Table 16. Annual flows of Power-to-SCP plants for 100 kt a^{-1} e-protein supply in 2030.

Item	Unit	CHL-PAT	AUS	US-CA	CHL-ATA	DEU	FIN	OMN
PV fixed tilted	GWh	263.1	2093.4	7479.1	6168.0	2661.5	1230.5	3666.2
PV single-axis	GWh	0.0	5174.9	0.0	1338.1	0.0	1472.7	3857.8
Wind power plant	GWh	7084.5	68.8	218.2	0.0	4701.3	5154.9	0.0
H_2-CCGT	GWh	91.7	0.1	0.0	0.0	0.1	29.6	0.0
H_2-OCGT	GWh	15.5	0.2	0.0	0.2	43.2	3.2	0.0
Battery interface	GWh_{in}	34.9	1388.1	1114.5	1260.5	245.3	245.7	1242.0
Battery storage	GWh_{out}	32.5	1290.9	1036.5	1172.2	228.1	228.5	1155.0
Curtailed electricity	GWh	183.9	241.0	529.6	359.1	255.7	792.8	416.1
Electrolyser	$GWh_{H2,HHV}$	3117.3	2902.5	2901.8	2902.4	3013.6	2966.8	2901.8
Replaced stack	$GWh_{H2,HHV}$	1146.3	21.7	0.1	93.5	767.4	740.0	146.7
H_2 compressor	$GWh_{H2,HHV}$	969.1	1598.0	1594.9	1626.3	1058.2	1033.6	1632.4
H_2 salt cavern	$GWh_{H2,HHV}$	0.0	1449.1	0.0	0.0	1057.8	0.0	0.0
H_2 rock cavern	$GWh_{H2,HHV}$	902.3	4.7	0.0	0.0	0.0	0.0	0.0
H_2 underground pipe	$GWh_{H2,HHV}$	66.8	144.2	1594.9	1626.3	0.4	1033.6	1632.4
O_2 processing unit	kt_{O2}	322.1	322.1	322.1	322.1	322.1	322.1	322.1
O_2 compressor	kt_{O2}	0.1	160.1	163.6	172.8	0.2	0.6	174.2
O_2 liquefaction	kt_{O2}	68.2	9.3	7.4	3.4	83.1	89.3	1.7
O_2 (g) storage	kt_{O2}	0.1	160.1	163.6	172.8	0.2	0.6	174.2
O_2 (l) storage	kt_{O2}	68.2	9.3	7.4	3.4	83.1	89.3	1.7
Direct air capture	kt_{CO2}	295.4	295.4	295.4	295.4	295.4	295.4	295.4
CO_2 compressor	kt_{CO2}	7.1	2.4	0.9	0.2	2.6	1.7	0.4
CO_2 liquefaction	kt_{CO2}	4.2	2.4	0.9	0.2	2.1	11.4	0.8
CO_2 (g) storage	kt_{CO2}	7.1	2.4	0.9	0.2	2.6	1.7	0.4
CO_2 (l) storage	kt_{CO2}	4.2	2.4	0.9	0.2	2.1	11.4	0.8
Heat pump	GWh_{th}	388.3	381.2	255.5	297.8	390.7	390.8	348.0
Electric water boiler	GWh_{th}	3.5	10.8	137.2	94.5	1.0	0.9	44.2
LT (78 °C) heat storage	$GWh_{th,in}$	283.1	277.9	186.3	217.1	284.8	284.9	253.7
HT (100 °C) heat storage	$GWh_{th,in}$	1.5	16.0	49.3	27.7	1.6	2.0	22.9
Electric steam boiler	GWh_{th}	165.3	108.5	64.0	91.9	148.3	112.8	104.3
Decoupled steam generator	GWh_{th}	750.9	807.7	852.1	824.2	767.8	803.3	811.8
Electric rod	GWh_{th}	807.1	867.6	914.7	885.1	822.2	866.5	871.3
VHT (300 °C) heat storage	$GWh_{th,in}$	807.1	867.6	914.7	885.1	822.2	866.5	871.3
Chiller	kt_{ChW}	273,037	273,051	273,037	273,039	273,038	273,043	273,037
Chilled Water storage	kt_{ChW}	19,941.6	1878.7	7110.9	1507.8	26,105.2	30,294.2	1,091.7
NH_3 plant	kt_{NH3}	24.0	24.0	24.0	24.0	24.0	24.0	24.0
NH_3 storage	kt_{NH3}	8.9	13.9	12.5	14.6	7.2	14.2	12.6
SCP core plant	kt_{SCP}	161.9	161.9	161.9	161.9	161.9	161.9	161.9
SCP storage	kt_{SCP}	10.8	5.1	11.9	5.6	6.4	10.4	5.3

Abbreviations: open cycle gas turbine (OCGT), closed cycle gas turbine (CCGT), low temperature (LT), high temperature (HT), and very high temperature (VHT).

Supplementary Table 17. Full load hours of components in Power-to-SCP plants for 100 kt a^{-1} e-protein supply in 2030.

	CHL-PAT	AUS	US-CA	CHL-ATA	DEU	FIN	OMN
PV fixed tilted	695	1723	1740	2015	967	980	1691
PV single-axis	-	2050	-	2356	1041	1109	1996
Wind power plant	5066	2201	2598	460	3472	3257	-
H_2-CCGT	1239	1044	1559	1735	1098	823	1888
H_2-OCGT	503	264	708	368	565	347	1002
Electrolyser	5061	3224	3057	3306	4293	4263	3370
H_2 compressor	3106	2604	2226	2627	2706	2187	2737
O_2 processing unit	5776	3490	3446	3485	5618	5226	3539
O_2 compressor	3398	2821	2818	2921	3051	3365	2935
O_2 liquefaction	4228	2939	2865	2535	4119	3873	2569
Direct air capture	7194	7720	6979	7483	7569	7317	7546
CO_2 compressor	1887	4026	2120	3319	1430	3422	2512
CO_2 liquefaction	2843	5784	3595	4348	2580	2215	4105
Heat pump	7741	8262	6732	7511	8121	7846	8136
Electric water boiler	4794	1961	3299	3938	1410	2672	2976
Electric steam boiler	2259	1748	830	1090	1506	1107	1354
Decoupled steam generator	6526	7347	6998	7256	6807	6824	7177
Electric rod	6011	2867	2512	2849	3210	3346	2976
Chiller	6719	7718	6828	7478	6323	6247	7529
NH_3 plant	6244	7284	5881	6092	7292	6103	6123
SCP core plant	7164	7769	6966	7483	7559	7242	7538

Abbreviations: open cycle gas turbine (OCGT) and closed cycle gas turbine (CCGT).

Supplementary Table 18. Capital cost of Power-to-SCP subunits for 100 kt a^{-1} e-protein supply in 2030.

Item	Unit	CHL-PAT	AUS	US-CA	CHL-ATA	DEU	FIN	OMN
PV fixed tilted	M€	115.9	371.8	1 315.1	936.9	842.0	384.1	663.3
PV single-axis	M€	0.0	850.6	0.0	191.4	0.0	447.6	651.3
Wind power plant	M€	1398.4	31.3	84.0	0.0	1354.1	1582.8	0.0
H_2-CCGT	M€	71.6	0.1	0.0	0.0	0.1	34.7	0.0
H_2-OCGT	M€	16.1	0.3	0.0	0.3	40.0	4.9	0.0
Battery interface	M€	3.9	49.6	42.4	46.0	12.5	10.4	40.8
Battery storage	M€	24.2	425.7	369.3	386.1	128.8	146.1	384.5
Electrolyser	M€	255.6	373.6	394.0	364.3	291.3	288.8	357.3
H_2 compressor	M€	16.5	32.5	38.0	32.8	20.7	25.0	31.6
H_2 salt cavern	M€	0.0	54.3	0.0	0.0	57.5	0.0	0.0
H_2 rock cavern	M€	161.6	0.4	0.0	0.0	0.0	0.0	0.0
H_2 underground pipe	M€	8.6	4.8	92.8	59.7	0.0	481.2	62.3
O_2 processing unit	M€	20.7	34.3	34.8	34.4	21.3	22.9	33.9
O_2 compressor	M€	0.0	12.9	13.2	13.5	0.0	0.0	13.5
O_2 liquefaction	M€	11.8	2.3	1.9	1.0	14.7	16.8	0.5
O_2 (g) storage	M€	0.0	23.1	24.1	24.4	0.0	0.1	25.2
O_2 (l) storage	M€	19.9	3.7	1.4	0.8	15.9	18.3	0.3
Direct air capture	M€	300.7	280.2	310.0	289.1	285.8	295.7	286.7
CO_2 compressor	M€	1.5	0.2	0.2	0.0	0.8	0.2	0.1
CO_2 liquefaction	M€	1.7	0.5	0.3	0.1	1.0	6.0	0.2
CO_2 (g) storage	M€	3.3	0.5	0.4	0.0	1.2	0.5	0.1
CO_2 (l) storage	M€	2.0	2.2	0.4	0.1	1.0	19.5	0.4
Heat pump	M€	32.6	30.0	24.7	25.8	31.3	32.4	27.8
Electric water boiler	M€	0.1	0.9	6.7	3.8	0.1	0.1	2.4
LT (78 °C) heat storage	M€	3.9	4.0	2.8	3.4	3.9	4.2	3.7
HT (100 °C) heat storage	M€	0.3	1.3	8.4	4.7	0.3	0.2	3.1
Electric steam boiler	M€	6.1	5.2	6.5	7.1	8.3	8.6	6.5
Decoupled steam generator	M€	8.7	8.4	9.3	8.6	8.6	8.9	8.6
Electric rod	M€	12.4	27.8	33.5	28.6	23.6	23.8	26.9
VHT (300 °C) heat storage	M€	88.7	73.5	80.6	75.0	69.2	111.8	73.2
Chiller	M€	40.6	35.4	40.0	36.5	43.2	43.7	36.3
Chilled water storage	M€	45.7	2.5	7.4	1.7	45.8	52.7	1.4
NH_3 plant	M€	30.0	25.7	31.8	30.7	25.7	30.7	30.6
NH_3 storage	M€	1.6	0.9	1.6	3.0	0.6	1.3	1.8
SCP core plant	M€	965.8	890.7	993.4	924.8	915.4	955.4	918.0
SCP storage	M€	2.2	1.0	3.8	1.7	1.3	2.4	1.6
Total	M€	3 673	3 662	3 973	3 536	4 266	5 062	3 694

Abbreviations: open cycle gas turbine (OCGT), closed cycle gas turbine (CCGT), low temperature (LT), high temperature (HT), and very high temperature heat (VHT).

Supplementary Table 19. Cost distribution of e-protein in 2030.

Item	Unit	CHL-PAT	AUS	US-CA	CHL-ATA	DEU	FIN	OMN
PV fixed tilted	€ $t^{-1}_{protein}$	113.1	362.9	1 283.5	914.3	821.7	374.9	647.3
PV single-axis	€ $t^{-1}_{protein}$	0.0	830.1	0.0	186.8	0.0	436.8	635.6
Wind power plant	€ $t^{-1}_{protein}$	1479.6	33.1	88.8	0.0	1432.8	1674.8	0.0
H_2-CCGT	€ $t^{-1}_{protein}$	75.0	0.1	0.0	0.0	0.1	36.1	0.0
H_2-OCGT	€ $t^{-1}_{protein}$	18.9	0.3	0.0	0.4	47.7	5.6	0.0
Battery interface	€ $t^{-1}_{protein}$	4.4	56.8	48.5	52.6	14.3	11.9	46.7
Battery storage	€ $t^{-1}_{protein}$	27.7	489.5	424.5	444.0	147.8	167.6	442.2
Electrolyser	€ $t^{-1}_{protein}$	329.7	463.8	487.3	453.0	369.9	366.4	444.9
Stack replacement	€ $t^{-1}_{protein}$	12.6	0.2	0.0	1.0	8.4	8.1	1.6
H_2 compressor	€ $t^{-1}_{protein}$	23.2	45.3	52.6	45.7	28.9	34.7	44.1
H_2 salt cavern	€ $t^{-1}_{protein}$	0.0	68.3	0.0	0.0	71.4	0.0	0.0
H_2 rock cavern	€ $t^{-1}_{protein}$	179.6	0.5	0.0	0.0	0.0	0.0	0.0
H_2 underground pipe	€ $t^{-1}_{protein}$	7.8	4.5	85.7	55.7	0.0	437.0	58.0
O_2 processing unit	€ $t^{-1}_{protein}$	23.3	38.3	38.8	38.3	23.9	25.7	37.8
O_2 compressor	€ $t^{-1}_{protein}$	0.0	17.6	18.0	18.3	0.0	0.1	18.4
O_2 liquefaction	€ $t^{-1}_{protein}$	14.9	2.9	2.4	1.3	18.6	21.3	0.6
O_2 (g) storage	€ $t^{-1}_{protein}$	0.0	22.2	23.2	23.5	0.0	0.1	24.2
O_2 (l) storage	€ $t^{-1}_{protein}$	19.1	3.5	1.3	0.8	15.3	17.6	0.3
Direct air capture	€ $t^{-1}_{protein}$	435.9	410.1	447.6	421.3	417.2	429.6	418.3
CO_2 compressor	€ $t^{-1}_{protein}$	2.1	0.3	0.2	0.0	1.0	0.3	0.1
CO_2 liquefaction	€ $t^{-1}_{protein}$	2.3	0.6	0.4	0.1	1.3	8.0	0.3
CO_2 (g) storage	€ $t^{-1}_{protein}$	3.1	0.5	0.3	0.0	1.1	0.5	0.1
CO_2 (l) storage	€ $t^{-1}_{protein}$	1.9	2.1	0.4	0.1	1.0	18.7	0.4
Heat pump	€ $t^{-1}_{protein}$	35.6	33.1	26.3	28.0	34.4	35.4	30.6
Electric water boiler	€ $t^{-1}_{protein}$	0.1	0.9	7.4	4.3	0.1	0.1	2.6
LT (78 °C) heat storage	€ $t^{-1}_{protein}$	3.7	3.8	2.7	3.2	3.7	4.0	3.5
HT (100 °C) heat storage	€ $t^{-1}_{protein}$	0.2	1.2	7.4	4.2	0.3	0.2	2.8
Electric steam boiler	€ $t^{-1}_{protein}$	6.5	5.4	6.4	7.0	8.4	8.5	6.5
Decoupled steam generator	€ $t^{-1}_{protein}$	9.7	9.2	10.2	9.5	9.5	9.9	9.5
Electric rod	€ $t^{-1}_{protein}$	11.2	25.2	30.3	25.9	21.3	21.6	24.4
VHT (300 °C) heat storage	€ $t^{-1}_{protein}$	81.2	67.4	73.9	68.8	63.5	102.1	67.2
Chiller	€ $t^{-1}_{protein}$	56.8	49.5	55.9	51.0	60.4	61.1	50.7
Chilled water storage	€ $t^{-1}_{protein}$	42.0	2.4	7.2	1.7	42.7	49.2	1.4
NH_3 plant	€ $t^{-1}_{protein}$	38.8	33.6	41.0	39.7	33.6	39.6	39.5
NH_3 storage	€ $t^{-1}_{protein}$	1.9	1.1	2.0	3.6	0.7	1.6	2.2
SCP core plant	€ $t^{-1}_{protein}$	1 426.6	1 325.4	1 463.7	1 371.3	1 358.7	1 412.6	1 362.2
SCP storage	€ $t^{-1}_{protein}$	2.8	1.3	4.6	2.1	1.6	3.1	1.9
Ramp up/down cost	€ $t^{-1}_{protein}$	0.8	0.7	1.0	0.8	0.6	0.7	0.7
Total	€ $t^{-1}_{protein}$	4 492	4 414	4 743	4 278	5 062	5 825	4 427

Supplementary Table 20. Required capacities of PV, wind power, battery, electrolyser, and DAC based on average of values for Power-to-SCP plants in Australia, Chile (Atacama Desert), and Germany.

	Unit	2035	2040	2045	2050	
Operational capacity	$Mt_{protein}$ a^{-1}	1.02	3.71	11.1	29.7	
SCP core plant's average FLh	h	7544	7538	7508	7495	
Annual supply	$Mt_{protein}$ a^{-1}	1.0	3.5	10.4	27.8	
Newly added supply	$Mt_{protein}$ $timestep^{-1}$	1.0	2.5	6.9	17.4	
Required capacities for 1 Mt a^{-1} e-protein supply						
PV	GW	33.6	33.3	32.8	32.3	
Wind	GW	4.1	3.6	3.4	3.2	
Battery interface	GW	6.8	5.5	5.4	5.3	
Battery Storage	GWh	30.0	26.9	26.1	25.4	
Electrolyser	$GW_{H2,HHV}$	8.0	8.4	8.3	8.3	
Direct air capture	Mt_{CO2} a^{-1}	3.4	3.4	3.5	3.5	
Required capacities for meeting the newly added supply at each timestep						total
PV	GW	33.6	83.1	226.3	562.4	905
Wind	GW	4.1	9.1	23.4	56.0	93
Battery interface	GW	6.8	13.9	37.3	92.3	150
Battery Storage	GWh	30.0	67.3	180.3	442.6	720
Electrolyser	$GW_{H2,HHV}$	8.0	20.9	57.6	144.1	231
Direct air capture	Mt_{CO2} a^{-1}	3.4	8.6	23.8	60.2	96

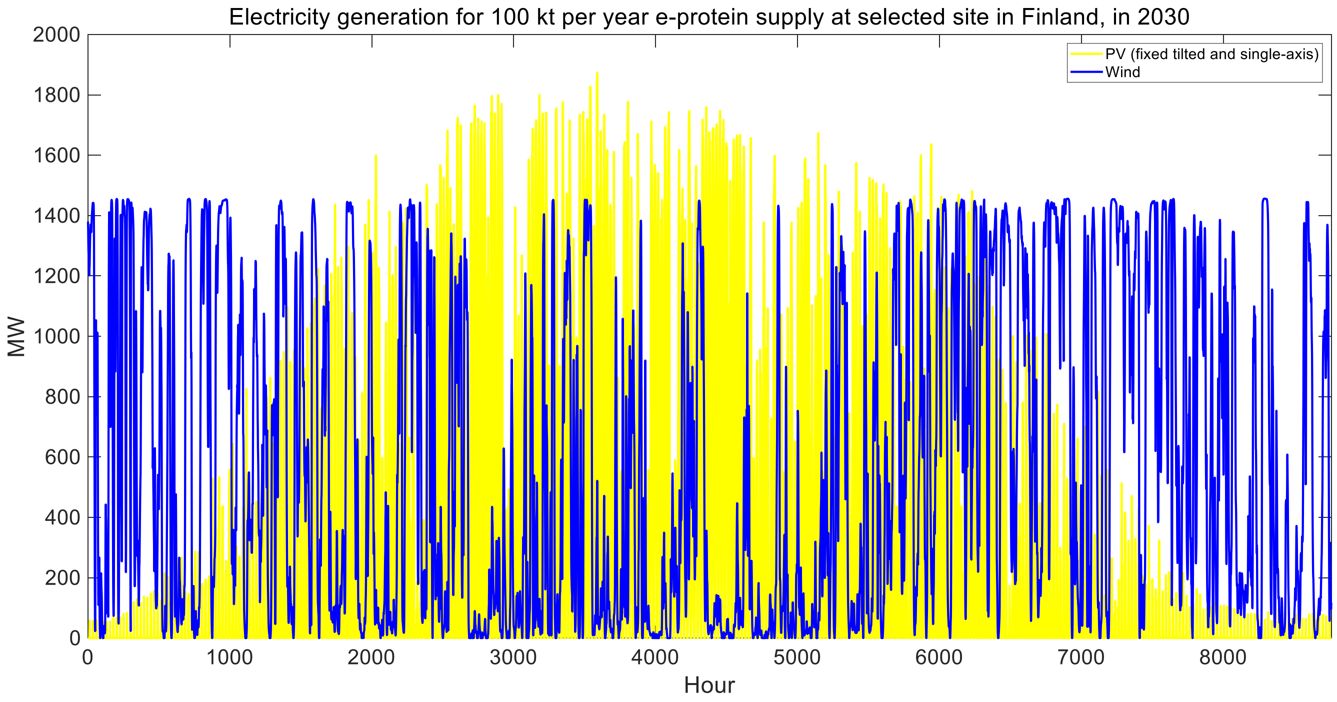

Supplementary Figure 7 | Electricity generation for 100 kt a^{-1} e-protein supply at a selected site in Finland, 2030. The optimal power generation system at the site in Finland includes both PV and wind power. Lower wind power generation in during the summer is balanced by higher PV generation.

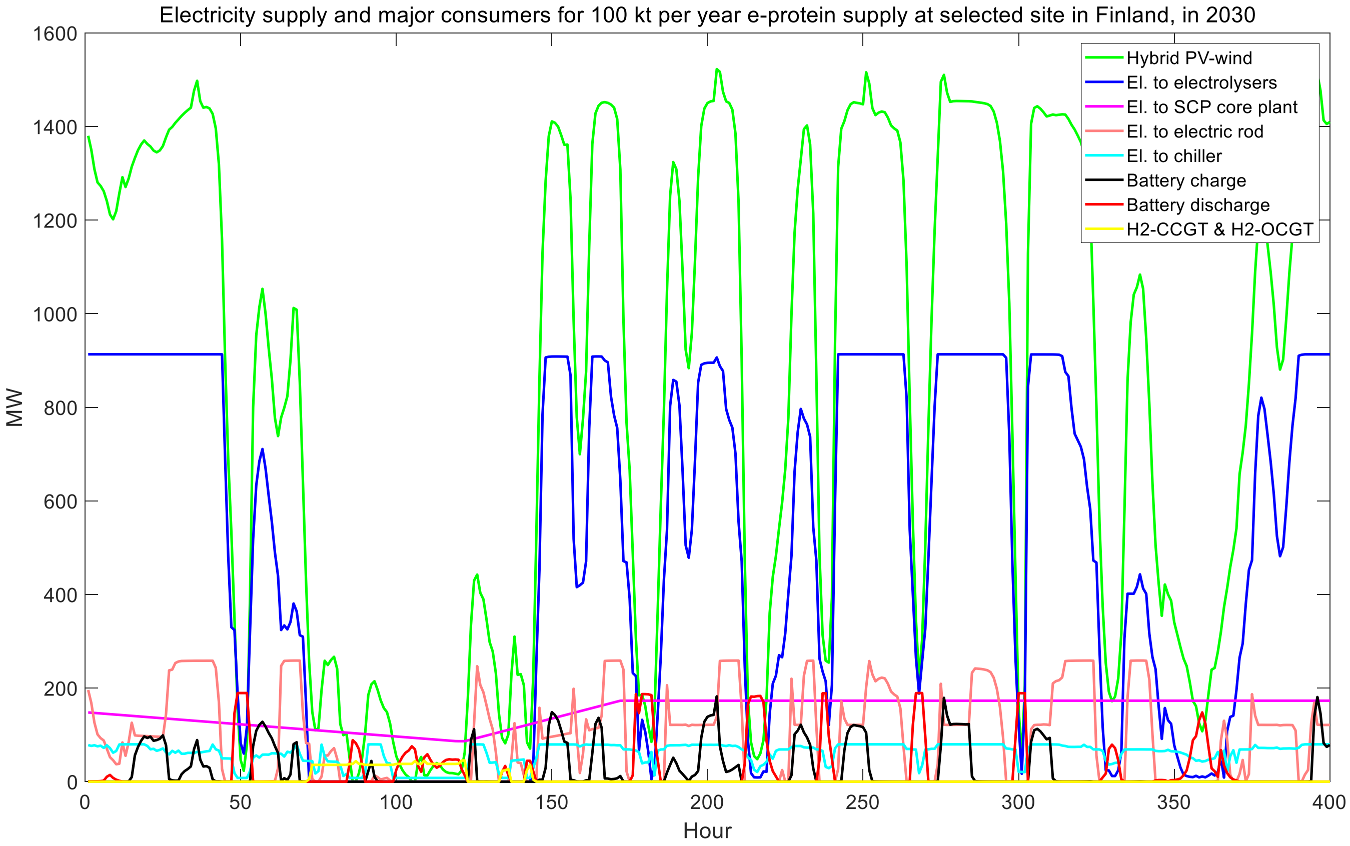

Supplementary Figure 8 | Electricity supply and major consumers for 100 kt a^{-1} e-protein supply at a selected site in Finland, 2030. The hourly electricity generation and consumption is shown for the first 400 hours of the year. Electrolysers and electric rods follow electricity generation profile. The utilisation rates of SCP core plant and chiller are extended by use of electricity from battery and H_2-fuelled gas turbines. In all hours, SCP core plant maintains a minimum operational load of 50% as a predefined system constraint. Relatively smaller electricity such as ammonia synthesis unit, electric steam boiler, electric water boiler, heat pump, DAC, and feed gases balancing units are not shown in the figure for better visualisation of key flows.

Supplementary Note 8. Sensitivity analyses

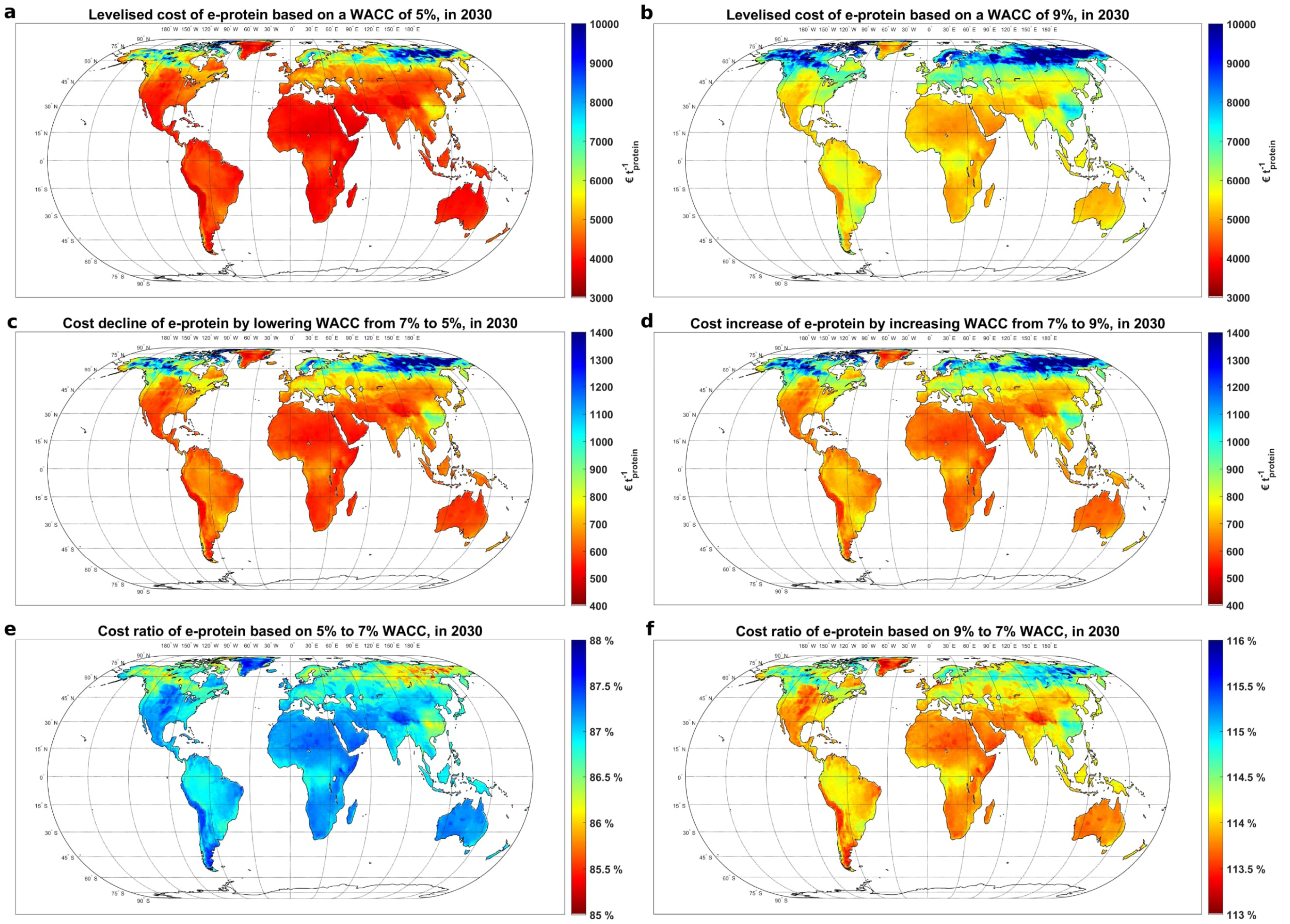

Supplementary Figure 9 | Impact of changes in WACC on e-protein production cost in 2030. a levelised cost of e-protein based on a WACC of 5%. **b** levelised cost of e-protein based on a WACC of 9%. **c** cost decline of e-protein by lowering WACC from 7% to 5%. **d** cost decline of e-protein by increasing WACC from 7% to 9%. **e** cost ratio of e-protein based on 5% to 7% WACC. **f** cost ratio of e-protein based on 9% to 7% WACC.

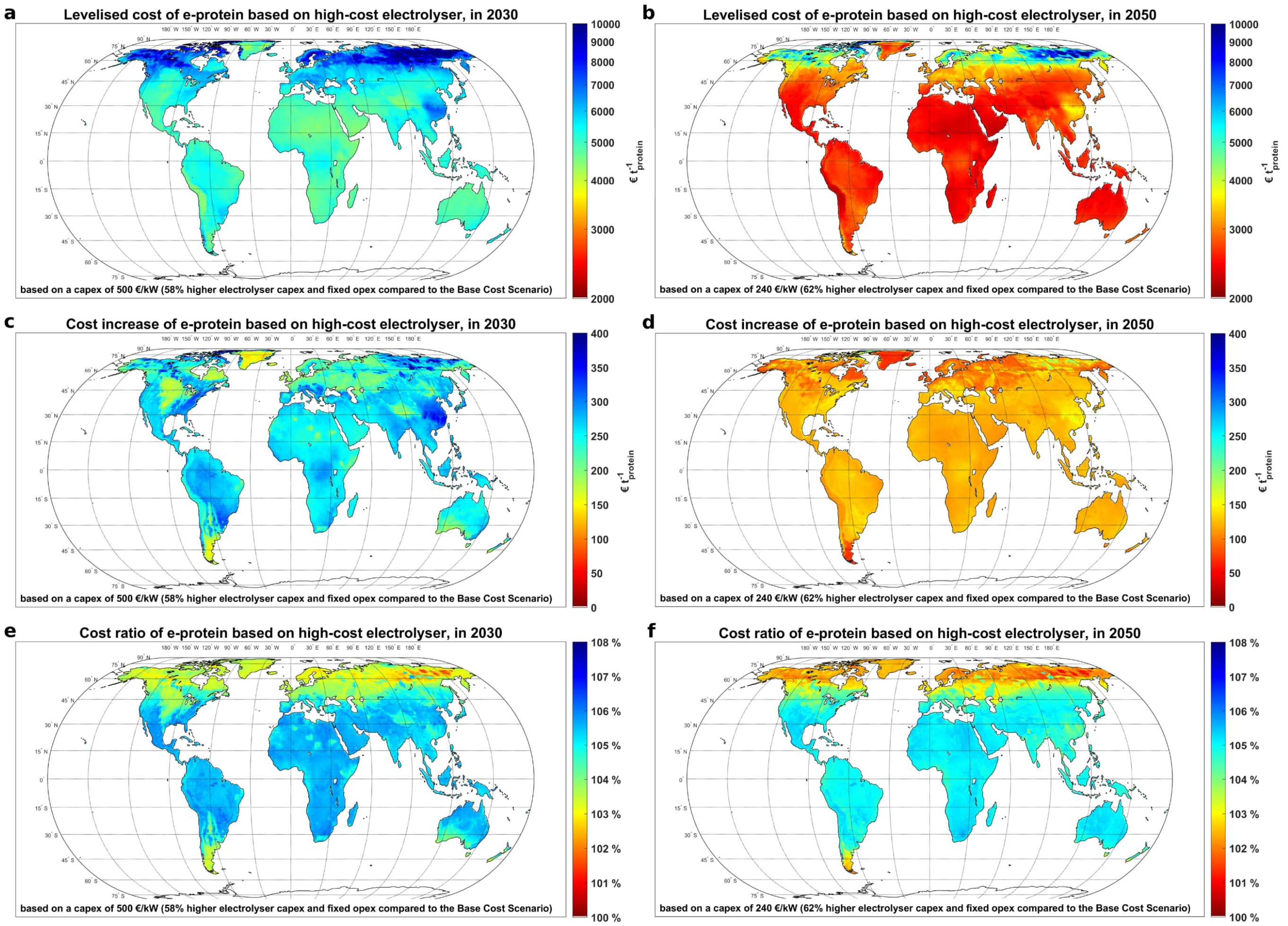

Supplementary Figure 10│Impact of the *High-Cost Scenario* for electrolyser capex and fixed opex on e-protein production cost. a levelised cost of e-protein based on high-cost electrolyser in 2030. **b** levelised cost of e-protein based on high-cost electrolyser in 2050. **c** cost increase of e-protein based on high-cost electrolyser in 2030. **d** cost increase of e-protein based on high-cost electrolyser in 2050. **e** cost ratio of e-protein based on high-cost to reference electrolyser in 2030. **f** cost ratio of e-protein based on high-cost to reference electrolyser in 2030.

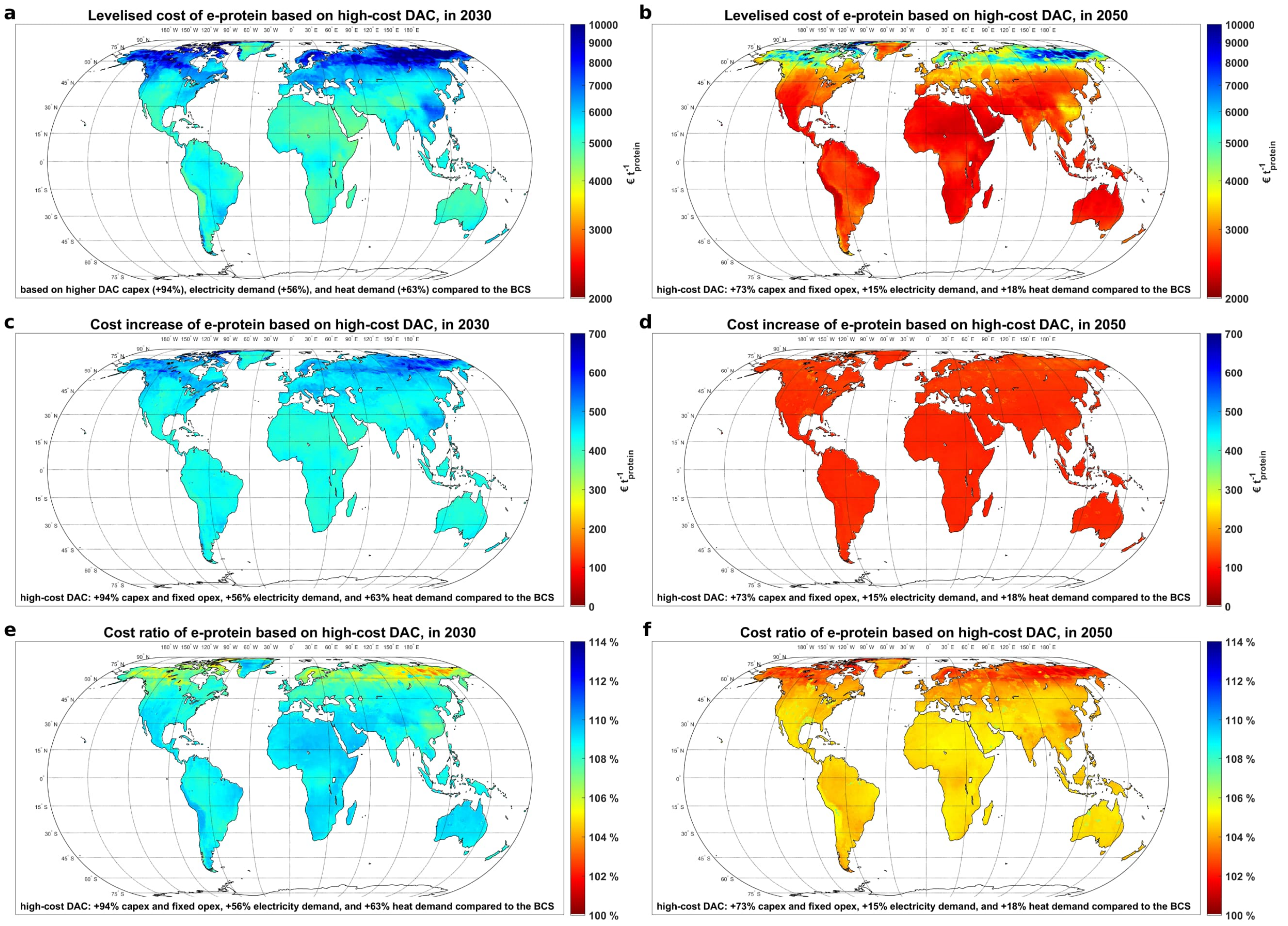

Supplementary Figure 11│Impact of the *High-Cost Scenario* for direct air capture (DAC) capex and fixed opex, and energy demand on e-protein production cost. a levelised cost of e-protein based on high-cost DAC in 2030. **b** levelised cost of e-protein based on high-cost DAC in 2050. **c** cost increase of e-protein based on high-cost DAC in 2030. **d** cost increase of e-protein based on high-cost DAC in 2050. **e** cost ratio of e-protein based on high-cost to reference DAC in 2030. **f** cost ratio of e-protein based on high-cost to reference electrolyser in 2030.

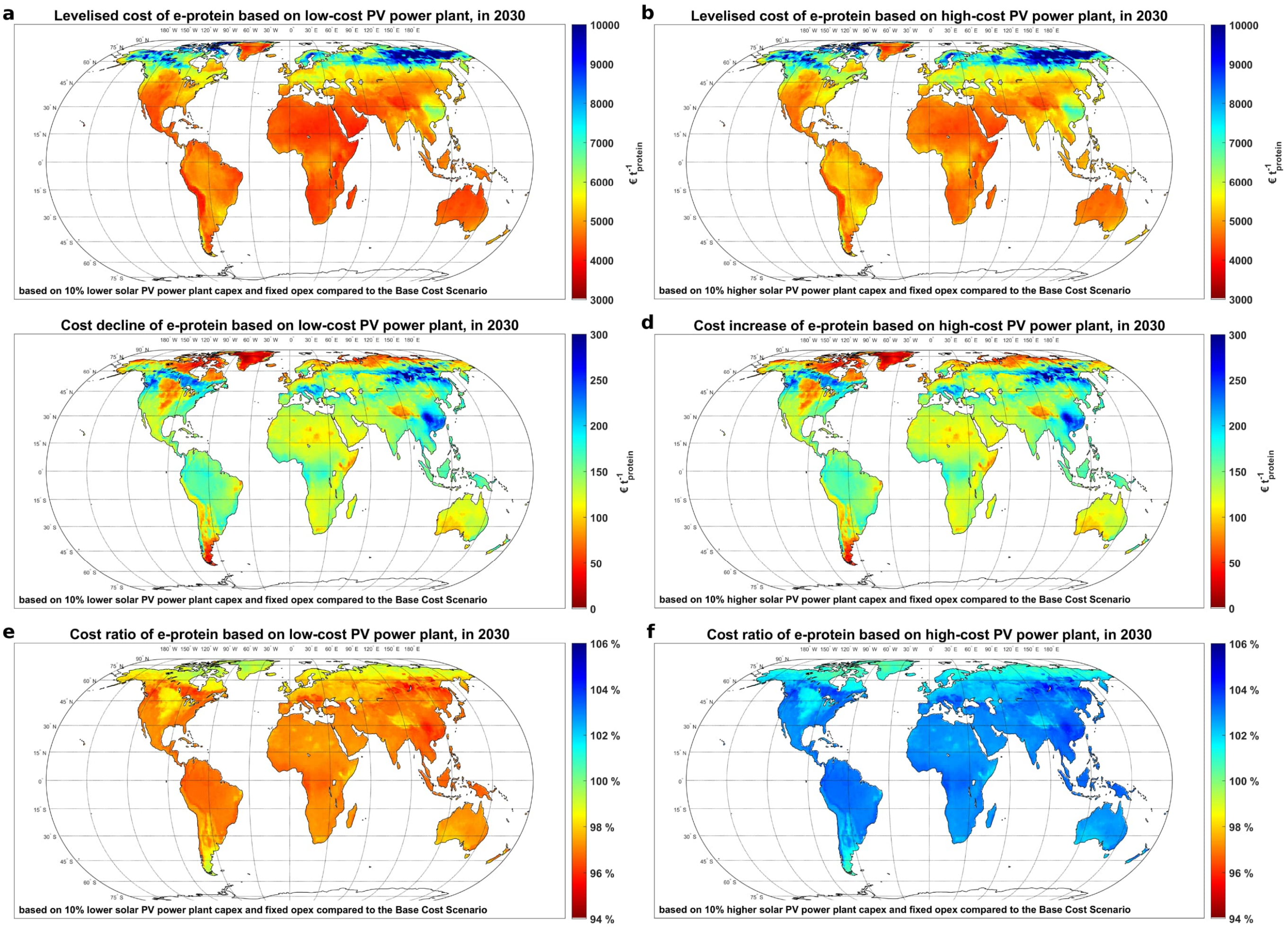

Supplementary Figure 12 | Impact of 10% change in the capex and fixed opex of solar PV on e-protein production cost in 2030. **a** levelised cost of e-protein based on low-cost PV power plant. **b** levelised cost of e-protein based on high-cost PV power plant. **c** cost decline of e-protein based on low-cost PV power plant. **d** cost increase of e-protein based on high-cost PV power plant. **e** cost ratio of e-protein based on low-cost to reference PV power plant. **f** cost ratio of e-protein based on high-cost to reference PV power plant.

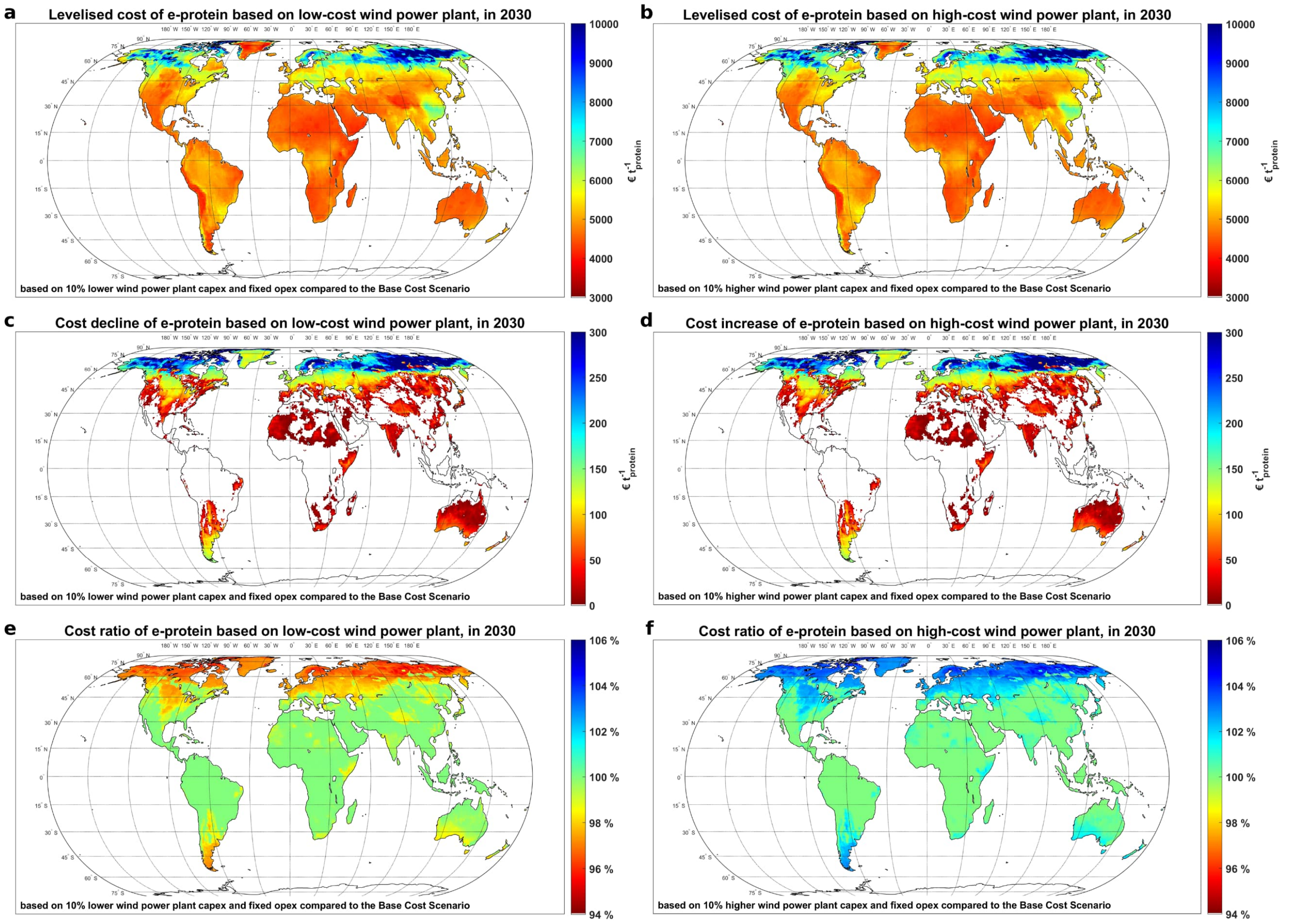

Supplementary Figure 13 | Impact of 10% change in the capex and fixed opex of wind power on e-protein production cost in 2030. **a** levelised cost of e-protein based on low-cost wind power plant. **b** levelised cost of e-protein based on high-cost wind power plant. **c** cost decline of e-protein based on low-cost wind power plant. **d** cost increase of e-protein based on high-cost wind power plant. **e** cost ratio of e-protein based on low-cost to reference wind power plant. **f** cost ratio of e-protein based on high-cost to reference wind power plant.

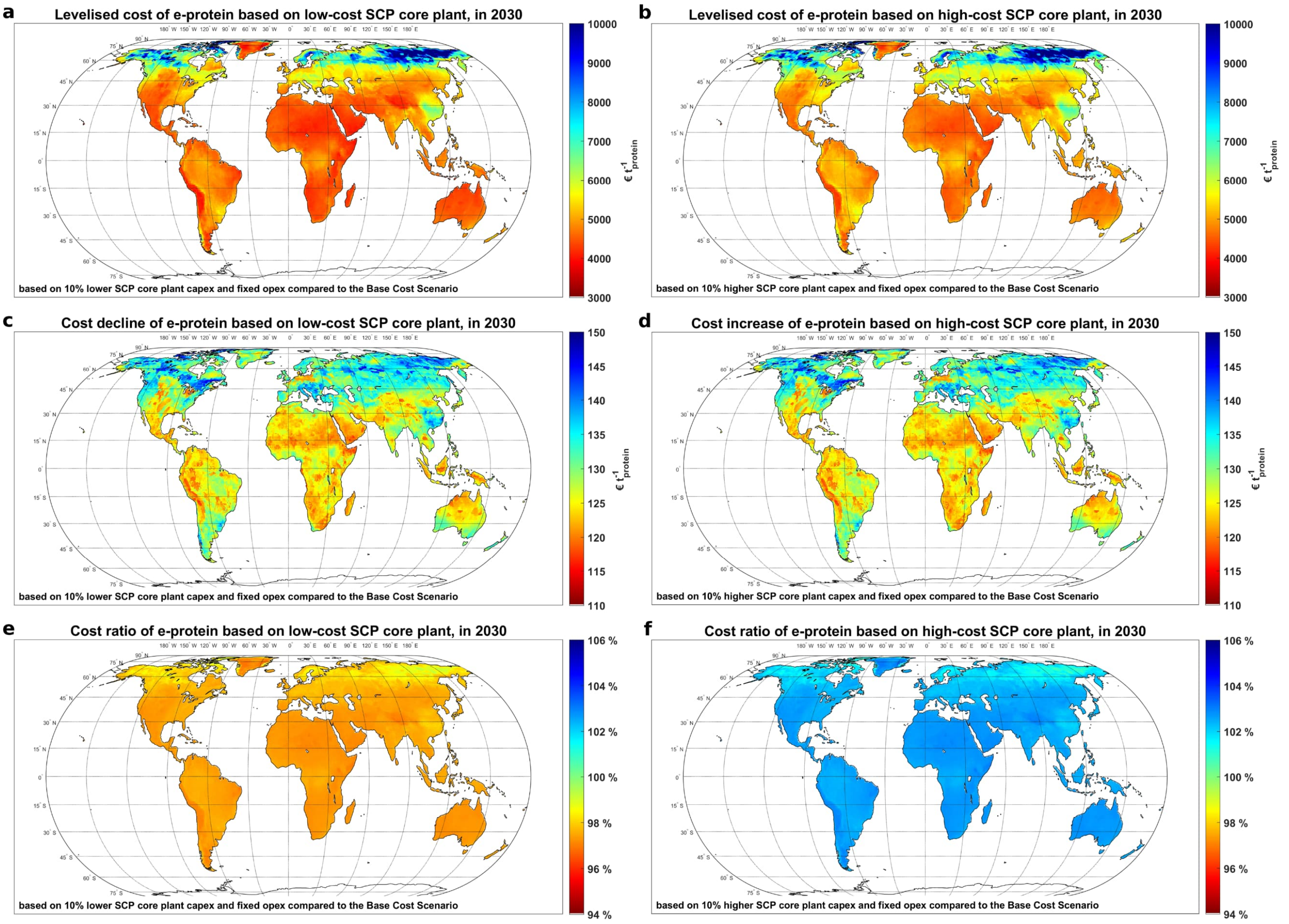

Supplementary Figure 14 | Impact of 10% change in the capex and fixed opex of SCP core plant on e-protein production cost in 2030. a levelised cost of e-protein based on low-cost SCP core plant. **b** levelised cost of e-protein based on high-cost SCP core plant. **c** cost decline of e-protein based on low-cost SCP core plant. **d** cost increase of e-protein based on high-cost SCP core plant. **e** cost ratio of e-protein based on low-cost to reference SCP core plant. **f** cost ratio of e-protein based on high-cost to reference SCP core plant.

Supplementary Note 9. Input data for optimisation

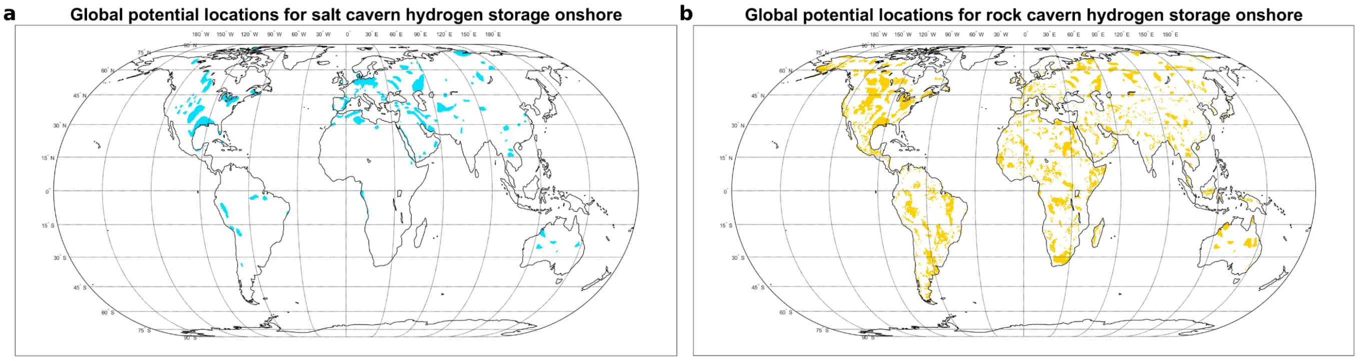

Supplementary Figure 15 │ Global potential locations for onshore geological hydrogen storage. a salt cavern. **b** rock cavern. Data adopted from [54].

Supplementary Table 21. Technical and financial specifications of applied technologies. Abbreviations: weighted average cost of capital (WACC), capital expenditures per unit of capacity (capex), annual operational expenditures per unit of capacity (opex $_{fix}$), variable operational costs (opex $_{var}$), efficiency (eff.), electricity (el.), learning rate (LR), working capacity (WC), full load hour (FLh), per annum (p. a.), lower heating value (LHV), higher heating value (HHV), base cost scenario (BCS), and high-cost scenario (HCS).

Item	Unit	2020/ref.	2025	2028	2030	2035	2040	2045	2050	Ref.
Globally uniform WACC	%	7	7	7	7	7	7	7	7	
PV fixed tilted power plant										
Capex	€ kW^{-1}_{p}	580	466		390	337	300	270	246	[55]
	€ kW^{-1}_{p}	432	336		278	237	207	184	166	[56]
	€ kW^{-1}_{p}	475	370	326	306	237	207	184	166	this study
Opex $_{fix}$	€ kW^{-1}_{p}	13.2			10.6		8.8		7.4	[55]
	€ kW^{-1}_{p}	7.76			5.66		4.47	4.04	3.7	[56]
	€ kW^{-1}_{p}	8.53	7.17	6.53	6.23	5	4.47	4.04	3.7	this study
Opex $_{var}$	€ kWh^{-1}_{el}	0	0	0	0	0	0	0	0	
Installation density	MW km^{-2}	91	100	106	109	118	127	137	137	based on [56,57]
Lifetime	year	30	35	35	35	35	40	40	40	[58]
PV single-axis tracking power plant										
Capex	€ kW^{-1}_{p}	638	513		429	371	330	297	271	[55,59]
	€ kW^{-1}_{p}	475	370		306	261	228	202	183	[56,59]
	€ kW^{-1}_{p}	523	407	359	337	261	228	202	183	this study
Opex $_{fix}$	€ kW^{-1}_{p}	15			12		10		8	[55,59]
	€ kW^{-1}_{p}	8.54			6.23		4.92		4.07	[56,59]
	€ kW^{-1}_{p}	9.4	7.88	7.17	6.86	5.5	4.92	4.44	4.07	this study
Opex $_{var}$	€ kWh^{-1}_{el}	0	0	0	0	0	0	0	0	
Installation density	MW km^{-2}	62	69	72	75	81	87	94	94	based on [56,57]
Lifetime	year	30	35	35	35	35	40	40	40	
Wind power plant (onshore)										
Capex	€ kW^{-1}_{p}	1150	1060	1030	1000	965	940	915	900	based on [60]
Opex fix	% of capex p.a.	2	2	2	2	2	2	2	2	
Opex var	€ kWh^{-1}_{el}	0	0	0	0	0	0	0	0	
Lifetime	year	25	25	25	25	25	25	25	25	
Installation density	MW km^{-2}	8.4	8.4	8.4	8.4	8.4	8.4	8.4	8.4	[61]
Disturbance factor	%	8	8	8	8	8	8	8	8	
Battery pack (storage) - lithium iron phosphate										
Capacity	MWh	80	80	80	80	80	80	80	80	[56]
Capex	€ kWh^{-1}	234	153	132	110	89	76	68	61	[56]
Opex fix	€ kWh^{-1}	3.28	2.6	2.40	2.2	2.05	1.9	1.77	1.71	[56]
Opex var	€ kWh^{-1}	0.0002	0.0002	0.0002	0.0002	0.0002	0.0002	0.0002	0.0002	

Item	Unit	2020/ref.	2025	2028	2030	2035	2040	2045	2050	Ref.
Lifetime	year	20	20	20	20	20	20	20	20	[62]
Cycle eff.	%	91	92	92.5	93	94	95	95	95	[63]
Self-discharge	% h^{-1}	0	0	0	0	0	0	0	0	
Battery interface (inverter, etc.)										
Capacity	MW	20	20	20	20	20	20	20	20	[56]
Capex	€ kW^{-1}	117	76	66	55	44	37	33	30	[56]
Opex $_{fix}$	€ kW^{-1}	1.64	1.29	1.20	1.10	1.01	0.93	0.86	0.84	[56]
Opex $_{var}$	€ kWh^{-1}	0	0	0	0	0	0	0	0	
Lifetime	year	20	20	20	20	20	20	20	20	[62]
Combined cycle gas turbine										
Capacity	MW	580	580	25	250	250	250	250	250	[64]
Capex (conventional) – 0.85 LR	€ kW^{-1}	775	775	1242	879	879	879	879	879	[64]
Capex (H_2-fuelled)	€ kW^{-1}		852.5	1366	967	967	967	967	967	10% higher
Opex $_{fix}$	% of capex p.a.		2.5	2.5	2.5	2.5	2.5	2.5	2.5	[64]
Opex $_{var}$	€ kWh^{-1}		0.002	0.002	0.002	0.002	0.002	0.002	0.002	[64]
Lifetime	year		35	35	35	35	35	35	35	[65]
Efficiency	% - LHV		61.2	61.2	61.2	62.3	63.3	63.3	63.3	[64]
Efficiency	% - HHV		52.2	52.2	52.2	53.1	54.0	54.0	54.0	
Open cycle gas turbine										
Capacity	MW	250	250	25	250	250	250	250	250	[64]
Capex (conventional) – 0.85 LR	€ kW^{-1}	475	475	475	475	475	475	475	475	[64]
Capex (H_2-fuelled)	€ kW^{-1}		523	738	523	523	523	523	523	10% higher
Opex $_{fix}$	% of capex p.a.		3.0	3.0	3.0	3.0	3.0	3.0	3.0	[64]
Opex $_{var}$	€ kWh^{-1}		0.011	0.011	0.011	0.011	0.011	0.011	0.011	[64]
Lifetime	year		35	35	35	35	35	35	35	[65]
Efficiency	% - LHV		43.8	44.47	45.4	45.9	46.5	47	47.5	
Efficiency	% - HHV		37.4	38.1	38.7	39.2	39.6	40.1	40.5	[64]
Alkaline water electrolyser										[66,67] & LR
Capacity	MW	28	250	100	250	250	250	250	250	
Capex (BCS)	€ kW^{-1}_{el}	638	446	377	316	234	189	163	148	
	€ $kW^{-1}_{H2,HHV}$		597	498	415	301	239	202	180	
Capex (HCS)	€ kW^{-1}_{el}		666	583	500	377	306	264	240	
	€ $kW^{-1}_{H2,HHV}$		891	771	656	485	387	327	292	
Opex $_{fix}$	% of capex p.a.	3.5	3.5	3.5	3.5	3.5	3.5	3.5	3.5	
Opex $_{var}$ – incl. water cons., excl. stack replacement	€ $kWh^{-1}_{H2,HHV}$	0.0011	0.0011	0.011	0.0011	0.0011	0.0011	0.0011	0.0011	
Stack replacement cost			40% of the electrolyser full system cost 15 years after initial installation							
Opex $_{var}$ – stack replacement cost (BCS)	€ $kWh^{-1}_{H2,HHV}$	0.0019	0.0014	0.0013	0.0011	0.0009	0.0009	0.0009	0.0009	

Item	Unit	2020/ref.	2025	2028	2030	2035	2040	2045	2050	Ref.
Opex $_{var}$ – stack replacement cost (HCS)	€ $kWh^{-1}_{H2,HHV}$	0.0031	0.0023	0.0021	0.0018	0.0015	0.0015	0.0015	0.0015	
Lifetime - system	year	30	30	30	30	30	30	30	30	
Lifetime - stack	hours	80 000	88 000	91 200	96 000	104 000	112 000	120 000	128 000	
Availability (single stack)	%	95	95	95	95	95	95	95	95	
Availability (system level)	%	100	100	100	100	100	100	100	100	
PtH_2 eff. - overall	% - LHV	62.5	63.8	64.3	65.0	66.3	67.5	68.8	70.0	
PtH_2 eff. - overall	% - HHV	73.3	74.8	75.3	76.2	77.7	79.1	80.6	82.1	
PtHeat eff.	% - utilisable	22.7	21.4	20.8	20.0	18.7	17.3	16.0	14.7	
Heat temperature	°C	75	75	75	75	75	75	75	75	
H_2 pressure	bar	5	30	30	30	30	30	30	30	
H_2 compressor										
Compression range	bar	30→150	30→150	30→150	30→150	30→150	30→150	30→150	30→150	
Electricity consumption	kWh_{el} $kWh^{-1}_{H2,HHV}$	0.025	0.025	0.025	0.025	0.025	0.025	0.025	0.025	[68]
	kWh_{el} kg^{-1}_{H2}	0.99	0.99	0.99	0.99	0.99	0.99	0.99	0.99	
Capacity	MW	1	1	1	3	3	3	3	3	[69]
	$MW_{H2,HHV}$	40	40	40	120	120	120	120	120	
	t_{H2} h^{-1}	1.01	1.01	1.01	3.04	3.04	3.04	3.04	3.04	
Capex	€ kW^{-1}	3 400	3 400	3 400	2 100	2 100	2 100	2 100	2 100	[69]
(scaling factor: 0.7)	€ $kW^{-1}_{H2,HHV}$	85	85	85	53	53	53	53	53	
	€ kg^{-1}_{H2} h	3 350	3 350	3 350	2 089	2 089	2 089	2 089	2 089	[61]
Opex $_{fix}$	% of capex p.a.		4.0	4.0	4.0	4.0	4.0	4.0	4.0	[70]
Opex $_{var}$	€ $kWh^{-1}_{H2,HHV}$		0.0001	0.0001	0.0001	0.0001	0.0001	0.0001	0.0001	
Lifetime	year		20	20	20	20	20	20	20	
Mass eff.	%		100	100	100	100	100	100	100	
Hydrogen storage - man-made salt cavern										
Working capacity (WC)	tonne H_2	500	500	500	2 000	2 000	2 000	2 000	2 000	[69]
	$GWh_{H2,HHV}$	20	20	20	79	79	79	79	79	
Capex - excluding cushion gas cost	€ kg^{-1}_{H2-WC}	24.9	24.9	24.9	16.4	16.4	16.4	16.4	16.4	[69]
(scaling factor: 0.7)	€ $kWh^{-1}_{H2,HHV}$	0.631	0.632	0.632	0.417	0.417	0.417	0.417	0.417	
Levelised cost of H_2 for cushion gas (global average)	€ $kWh^{-1}_{H2,HHV\text{ - produced}}$	0.071	0.053	0.046	0.042	0.031	0.027	0.023	0.020	based on [71]
Cushion gas cost - *(cushion/total capacity: 0.3)*	€ $kWh^{-1}_{H2,HHV\text{ - WC}}$	0.032	0.024	0.021	0.019	0.015	0.012	0.011	0.009	
Capex - including cushion gas cost	€ kg^{-1}_{H2-WC}	26.2	25.9	25.7	17.2	17.0	16.9	16.9	16.8	
	€ $kWh^{-1}_{H2,HHV}$	0.664	0.656	0.653	0.436	0.432	0.429	0.428	0.426	
Opex $_{fix}$	% of capex p.a.	4.0	4.0	4.0	4.0	4.0	4.0	4.0	4.0	[72]
Opex $_{var}$	€ $kWh^{-1}_{H2,HHV}$	0.0002	0.0002	0.0002	0.0002	0.0002	0.0002	0.0002	0.0002	
Lifetime	year	30	30	30	30	30	30	30	30	[72]
Cycle eff.	%	100	100	100	100	100	100	100	100	

Item	Unit	2020/ref.	2025	2028	2030	2035	2040	2045	2050	Ref.
Self-discharge	% h^{-1}	0	0	0	0	0	0	0	0	
Maximum charge rate	% day^{-1}	10	10	10	10	10	10	10	10	[73]
Minimum Energy-to-Power ratio	h	240	240	240	240	240	240	240	240	
Pressure range	bar	43–150	43–150	43–150	43–150	43–150	43–150	43–150	43–150	[69]
Hydrogen storage - lined rock cavern										
Working capacity (WC)	tonne H_2	100	100	100	500	500	500	500	500	[69]
	$GWh_{H2,HHV}$	3.9	3.9	3.9	20	20	20	20	20	
Capex - excluding cushion gas - *(scaling factor: 0.8)*	€ kg^{-1}_{H2}	79	79	79	57	57	57	57	57	[69]
	€ $kWh^{-1}_{H2,HHV}$	1.440	1.730	1.730	1.254	1.254	1.254	1.254	1.254	
Levelised cost of H_2 for cushion gas (globally unified)	€ $kWh^{-1}_{H2,HHV\text{ - produced}}$	0.071	0.053	0.046	0.042	0.031	0.027	0.023	0.020	based on [71]
Cushion gas cost - *(cushion/total capacity: 0.144)*	€ $kWh^{-1}_{H2,HHV\text{ - WC}}$	0.013	0.010	0.008	0.008	0.006	0.005	0.004	0.004	
Capex - including cushion gas cost	€ $kWh^{-1}_{H2,HHV}$	2.000	1.997	1.995	1.448	1.446	1.445	1.444	1.444	
Opex $_{fix}$	% of capex p.a.	3.0%	3.0%	3.0%	3.0%	3.0%	3.0%	3.0%	3.0%	
Opex $_{var}$	€ $kWh^{-1}_{H2,HHV}$	0.0001	0.0001	0.0001	0.0001	0.0001	0.0001	0.0001	0.0001	
Lifetime	year	30	30	30	30	30	30	30	30	
Cycle eff.	%	100	100	100	100	100	100	100	100	
Self-discharge	% h^{-1}	0	0	0	0	0	0	0	0	
Maximum charge rate	% day^{-1}	10	10	10	10	10	10	10	10	
Minimum Energy-to-Power ratio	h	240	240	240	240	240	240	240	240	
Pressure range	bar	20–150	20–150	20–150	20–150	20–150	20–150	20–150	20–150	[69]
Hydrogen storage - underground pipe										
Working capacity (WC)	tonne H2	25	25	25	250	250	250	250	250	[69]
	$GWh_{H2,HHV}$	1.0	1.0	1.0	9.9	9.9	9.9	9.9	9.9	
Capex	€ kg^{-1}_{H2}	492	492	492	459	459	459	459	459	[69]
(scaling factor: 0.97)	€ $kWh^{-1}_{H2,HHV}$	12.5	12.5	12.5	11.6	11.6	11.6	11.6	11.6	
Opex $_{fix}$	% of capex p.a.	1.0%	1.0%	1.0%	1.0%	1.0%	1.0%	1.0%	1.0%	
Opex $_{var}$	€ $kWh^{-1}_{H2,HHV}$	0.0001	0.0001	0.0001	0.0001	0.0001	0.0001	0.0001	0.0001	
Lifetime	year	30	30	30	30	30	30	30	30	
Cycle eff.	%	100	100	100	100	100	100	100	100	
Self-discharge	% h^{-1}	0	0	0	0	0	0	0	0	
Minimum Energy-to-Power ratio	h	6	6	6	6	6	6	6	6	
Maximum charge rate	% h^{-1}	16.67	16.67	16.67	16.67	16.67	16.67	16.67	16.67	
Pressure range	bar	8-100	8-100	8-100	8-100	8-100	8-100	8-100	8-100	[69]
NH_3 Plant - including Air Separation Unit, N_2 & H_2 compressors, and ammonia synthesis unit										[74–76]
Capacity	t_{NH3} h^{-1}	2.3	0.3	0.3	3	3	3	3	3	
Capex - *(scaling factor: 0.78)*	€ $kg^{-1}_{NH3}\cdot h$	8 300	12 960	12 960	7 810	7 810	7 810	7 810	7 810	
Opex $_{fix}$	% of capex p.a.	4.0	4.0	4.0	4.0	4.0	4.0	4.0	4.0	

Item	Unit	2020/ref.	2025	2028	2030	2035	2040	2045	2050	Ref.
Opex $_{var}$	€ t^{-1}_{NH3}	11	11	11	11	11	11	11	11	
Lifetime	year	30	30	30	30	30	30	30	30	
Electricity consumption	kWh t^{-1}_{NH3}	738	738	738	738	738	738	738	738	
minimum load	% of full capacity	25	25	25	25	25	25	25	25	
Ramp-up time (min to max load)	% h^{-1}	2	2	2	2	2	2	2	2	
Ramp-down time (max to min load)	% h^{-1}	20	20	20	20	20	20	20	20	
Ramp-up/down cost	€ t^{-1}_{NH3} h^2	2	2	2	2	2	2	2	2	
Availability	h	8000	8000	8000	8000	8000	8000	8000	8000	
pressure	bar	150	150	150	150	150	150	150	150	
H_2 & N_2 conversion rate	%	99	99	99	99	99	99	99	99	
H_2 consumption	kg_{H2} t^{-1}_{NH3}	179.3	179.3	179.3	179.3	179.3	179.3	179.3	179.3	
N_2 consumption	kg_{N2} t^{-1}_{NH3}	830.9	830.9	830.9	830.9	830.9	830.9	830.9	830.9	
NH_3 storage										[76]
Capacity	t_{NH3}	3000	200	200	2000	2000	2000	2000	2000	
Capex (via 0.8 scaling factor)	€ t^{-1}_{NH3}	990	1800	1800	1080	1080	1080	1080	1080	
Opex $_{fix}$	% of capex p.a.	4	4	4	4	4	4	4	4	
Opex $_{var}$	€ t^{-1}_{NH3}	0.2	0.2	0.2	0.2	0.2	0.2	0.2	0.2	
Lifetime	year	30	30	30	30	30	30	30	30	
Minimum Energy-to-Power ratio	h	168	168	168	168	168	168	168	168	
Cycle eff.	%	100	100	100	100	100	100	100	100	
Self-discharge	% h^{-1}	0	0	0	0	0	0	0	0	
Low-Temperature CO_2 Direct Air Capture at 1 bar										section 6 & [52]
Capacity	kt_{CO2} a^{-1} – 8000 FLh	4	36	36	360	360	360	360	360	
	t_{CO2} h^{-1}	0.5	4.5	4.5	45	45	45	45	45	
Capex (BCS)	€ t^{-1}_{CO2} a – 8760 FLh	2 378	2 378	1 321	810	561	417	352	315	
	€ kg^{-1}_{CO2} h	20 833	20 833	11 569	7 096	4 914	3 653	3 084	2 759	
Capex (HCS)	€ t^{-1}_{CO2} a – 8760 FLh				1594				563	
Opex $_{fix}$	% of capex p.a.	4.0	4.0	4.0	4.0	4.0	4.0	4.0	4.0	
Opex $_{var}$	€ t^{-1}_{CO2}	32.0	32.0	23.9	19.5	14.7	11.7	10.3	9.5	
of which sorbent	€ t^{-1}_{CO2}	31	31	22.9	18.5	13.7	10.7	9.3	8.5	
of which others	€ t^{-1}_{CO2}	1	1	1	1	1	1	1	1	
Lifetime	year	20	25	25	25	30	30	30	30	
Availability	%	91.3	91.3	91.3	91.3	91.3	91.3	91.3	91.3	
Output pressure	bara	1	1	1	1	1	1	1	1	
Electricity demand (BCS)	kWh_{el} t^{-1}_{CO2}	700	700	336	309	275	250	237	229	
Electricity demand (HCS)	kWh_{el} t^{-1}_{CO2}				505				269	
LT heat demand (BCS)	kWh_{th} t^{-1}_{CO2}	3000	3000	1440	1326	1179	1072	1016	981	

Item	Unit	2020/ref.	2025	2028	2030	2035	2040	2045	2050	Ref.
LT heat demand (HCS)	$kWh_{th}\ t^{-1}_{CO2}$				2163				1154	
Heat temperature	°C	100	100	100	100	100	100	100	100	
CO_2 compressor #1 (coupled to DAC)										
Pressure range	bar	1→5	1→5	1→5	1→5	1→5	1→5	1→5	1→5	
Electricity consumption	$kWh\ t^{-1}_{CO2}$	40	40	40	40	40	40	40	40	[22]
Unit capacity	$t_{CO2}\ h^{-1}$	0.5	4.5	4.5	10	10	10	10	10	
	kW_{el}	20	180	180	400	400	400	400	400	
Capex	€ kW^{-1}_{el}	19 300	10 000	10 000	5 800	5 800	5 800	5 800	5 800	[23]
	€ $kg^{-1}_{CO2}\ h$	772	400	400	232	232	232	232	232	
Opex $_{fix}$	% of capex p.a.	4.0	4.0	4.0	4.0	4.0	4.0	4.0	4.0	
Opex $_{var\ (excl.\ el.)}$	€ t^{-1}_{CO2}	0.001	0.001	0.001	0.001	0.001	0.001	0.001	0.001	
Lifetime	year	20	20	20	20	20	20	20	20	
Low-Temperature DAC farm coupled to 1-5 bar CO_2 compressor										this study
Capacity	$t_{CO2}\ h^{-1}$	0.5	4.5	4.5	45	45	45	45	45	
Capex (BCS)	€ t^{-1}_{CO2} a – 8760 FLh	2 466	2 424	1 367	836	587	443	378	341	
	€ $kg^{-1}_{CO2}\ h$	21 604	21 236	11 972	7 323	5 142	3 881	3 311	2 987	
Capex (HCS)	€ t^{-1}_{CO2} a – 8760 FLh				1 620				589	
	€ $kg^{-1}_{CO2}\ h$				14 192				5 155	
Opex $_{fix}$	% of capex p.a.	4.0	4.0	4.0	4.0	4.0	4.0	4.0	4.0	
Opex $_{var}$	€ t^{-1}_{CO2}	32.0	32.0	23.9	19.5	14.7	11.7	10.3	9.5	
Electricity demand (BCS)	$kWh_{el}\ t^{-1}_{CO2}$	740	740	376	349	315	290	277	269	
Electricity demand (HCS)	$kWh_{el}\ t^{-1}_{CO2}$				545				309	
LT heat demand (BCS)	$kWh_{th}\ t^{-1}_{CO2}$	3 000	3 000	1 440	1 326	1 179	1 072	1 016	981	
LT heat demand (HCS)	$kWh_{th}\ t^{-1}_{CO2}$				2 163				1 154	
Availability	%	91.3	91.3	91.3	91.3	91.3	91.3	91.3	91.3	
Lifetime	year	20	25	25	25	30	30	30	30	
CO_2 compressor #2 (prior to CO_2 storage)										
Pressure range	bara	5→15	5→15	5→15	5→50	5→50	5→50	5→50	5→50	
Electricity consumption	$kWh\ t^{-1}_{CO2}$	27	27	27	56	56	56	56	56	[22]
Unit capacity	$t_{CO2}\ h^{-1}$	1	1	1	5	5	5	5	5	
	kW_{el}	27	27	27	280	280	280	280	280	
Capex	€ kW^{-1}_{el}	397	397	397	409	409	409	409	409	[23] and SF
	€ $kg^{-1}_{CO2}\ h$	45	45	45	47	47	47	47	47	
Opex $_{fix}$	% of capex p.a.	4.0	4.0	4.0	4.0	4.0	4.0	4.0	4.0	
Opex $_{var\ (excl.\ el.)}$	€ t^{-1}_{CO2}	0.1	0.1	0.1	0.1	0.1	0.1	0.1	0.1	
Lifetime	year	20	20	20	20	20	20	20	20	
CO_2 liquefaction plant										[66]

Item	Unit	2020/ref.	2025	2028	2030	2035	2040	2045	2050	Ref.
Unit capacity	$t_{CO2}\ h^{-1}$	1	1	1	5	5	5	5	5	
Feed gas pressure	bar	5	5	5	5	5	5	5	5	
Capex	€ kg^{-1}_{CO2} h	1880	1880	1880	1160	1160	1160	1160	1160	
	€ t^{-1}_{CO2} a	215	215	215	132	132	132	132	132	
Opex $_{fix}$	% of capex p.a.	4.0	4.0	4.0	4.0	4.0	4.0	4.0	4.0	
Opex $_{var\ (excl.\ el.)}$	€ t^{-1}_{CO2}	0.1	0.1	0.1	0.1	0.1	0.1	0.1	0.1	
Lifetime	year	20	20	20	20	20	20	20	20	
Electricity consumption	kWh t^{-1}_{CO2}	150	150	150	150	150	150	150	150	
Ramp-up time	% h^{-1}	100	100	100	100	100	100	100	100	
CO_2 (g) storage										66
Operating pressure range	bar	5-15	5-15	5-15	20-50	20-50	20-50	20-50	20-50	
Unit capacity	t_{CO2}	160	160	160	1 600	1 600	1 600	1 600	1 600	
Capex	€ t^{-1}_{CO2}	28 000	28 000	28 000	22 000	22 000	22 000	22 000	22 000	
Opex fix	% of capex p.a.	1.5	1.5	1.5	1.3	1.3	1.3	1.3	1.3	
Opex var	€ t^{-1}_{CO2}	0.1	0.1	0.1	0.1	0.1	0.1	0.1	0.1	
Lifetime	year	30	30	30	30	30	30	30	30	
Minimum charge/discharge time	h	6	6	6	6	6	6	6	6	
Cycle eff.	%	100	100	100	100	100	100	100	100	
Self-discharge	% h^{-1}	0	0	0	0	0	0	0	0	
CO_2 (l) storage tank										66
Unit capacity	t_{CO2}	560	560	560	560	560	560	560	560	
Capex	€ t^{-1}_{CO2}	7 120	7 120	7 120	7 120	7 120	7 120	7 120	7 120	
Opex fix	% of capex p.a.	1.5	1.5	1.5	1.5	1.5	1.5	1.5	1.5	
Opex var	€ t^{-1}_{CO2}	0.2	0.2	0.2	0.2	0.2	0.2	0.2	0.2	
Lifetime	year	30	30	30	30	30	30	30	30	
Minimum charge/discharge time	h	6	6	6	6	6	6	6	6	
Cycle eff.	%	100	100	100	100	100	100	100	100	
Self-discharge	% h^{-1}	0	0	0	0	0	0	0	0	
Heat pump - electrical compression										77
Unit capacity	MWh_{th}	3	3	3	3	3	3	3	3	
Capex	€ kW^{-1}_{th}	730	682	661	650	620	600	588	580	
Opex $_{fix}$	€ kW^{-1}_{th} p.a.	2	2	2	2	2	2	2	2	
Opex $_{var}$	€ kWh^{-1}_{th}	0.00180	0.00174	0.00171	0.00170	0.00170	0.00170	0.00164	0.00160	
Lifetime	year	20	20	20	20	20	20	20	20	
COP (soure@40C, sink@78-100C)	-	3.5	3.5	3.5	3.5	3.5	3.5	3.5	3.5	
Availability	%	98.1	98.1	98.1	98.1	98.1	98.1	98.1	98.1	
Electric water boiler										77

Item	Unit	2020/ref.	2025	2028	2030	2035	2040	2045	2050	Ref.
Unit capacity	MWh_{th}	2	2	2	2	2	2	2	2	
Capex	€ kW^{-1}_{th}	186	170	163	160	160	160	160	160	
Opex $_{fix}$	€ kW^{-1}_{th} p.a.	1.070	1.040	1.027	1.020	0.990	0.970	0.940	0.920	
Opex $_{var}$	€ kWh^{-1}_{th}	0.00050	0.00049	0.00048	0.00048	0.00046	0.00045	0.00044	0.00043	
Lifetime	year	25	25	25	25	25	25	25	25	
Efficiency	%	99	99	99	99	99	99	99	99	
Availability	h	8558	8558	8558	8558	8558	8558	8558	8558	
Electric steam boiler (12 bar)										66
Unit capacity	MWh_{th}	2	2	2	15	15	15	15	15	
Capex	€ kW^{-1}_{th}	213	197	190	84	84	84	84	84	
Opex $_{fix}$	€ kW^{-1}_{th} p.a.	1.221	1.192	1.179	1.056	1.025	1.004	0.973	0.952	
Opex $_{var}$	€ kWh^{-1}_{th}	0.00058	0.00056	0.00055	0.00049	0.00048	0.00047	0.00045	0.00044	
Lifetime	year	25	25	25	25	25	25	25	25	
Efficiency (power-to-heat)	%	99	99	99	99	99	99	99	99	
Efficiency (power-to-utilisable steam) - based on input water temp.	%			94.8	94.8	95.0	95.2	95.4	95.6	
Availability	%	97.7	97.7	97.7	97.7	97.7	97.7	97.7	97.7	
Low-Temperature Heat Storage (hot water storage tank, used for a temperature range of 20–78 °C)										
Unit capacity	tonne water	43 000	742	742	4 450	4 450	4 450	4 450	4 450	
	MWh_{th}		50	50	300	300	300	300	300	
Capex (via 0.8 Scaling Factor)	€ t^{-1}_{H2O}	628	1415	1415	989	989	989	989	989	78
	€ kWh^{-1}_{th}		21.0	21.0	14.7	14.7	14.7	14.7	14.7	
Opex $_{fix}$	% of capex p.a.	0.75	0.75	0.75	0.75	0.75	0.75	0.75	0.75	79
Opex $_{var}$	€ kWh^{-1}_{th}	0.0001	0.0001	0.0001	0.0001	0.0001	0.0001	0.0001	0.0001	
Lifetime	year	30	30	30	30	30	30	30	30	79
Cycle eff.	%	98.0	98.0	98.0	98.0	98.0	98.0	98.0	98.0	self-assumption
Energy-to-Power ratio	h	7	7	7	7	7	7	7	7	78
Self-discharge	% day^{-1}	0.12	0.12	0.12	0.12	0.12	0.12	0.12	0.12	78
Self-discharge	% h^{-1}	0.99995	0.99995	0.99995	0.99995	0.99995	0.99995	0.99995	0.99995	
Medium-Temperature Heat Storage (hot water storage tank, used for a temperature range of 78–98 °C)										
Unit capacity	tonne water	43 000	2150	2 150	8 600	8 600	8 600	8 600	8 600	
	MWh_{th}		50	50	200	200	200	200	200	
Capex	€ t^{-1}_{H2O}	628	1 143	1 143	866	866	866	866	866	78
	€ kWh^{-1}_{th}		49.2	49.2	37.3	37.3	37.3	37.3	37.3	
Opex $_{fix}$	% of capex p.a.	0.75	0.75	0.75	0.75	0.75	0.75	0.75	0.75	79
Opex $_{var}$	€ kWh^{-1}_{th}	0.0001	0.0001	0.0001	0.0001	0.0001	0.0001	0.0001	0.0001	
Lifetime	year	30	30	30	30	30	30	30	30	79

Item	Unit	2020/ref.	2025	2028	2030	2035	2040	2045	2050	Ref.
Cycle efficiency	%	98.0	98.0	98.0	98.0	98.0	98.0	98.0	98.0	self-assumption
Energy-to-Power ratio	-	7	7	7	7	7	7	7	7	[78]
Self-discharge	% day^{-1}	0.12	0.12	0.12	0.12	0.12	0.12	0.12	0.12	[78]
Self-discharge	% h^{-1}	0.99995	0.99995	0.99995	0.99995	0.99995	0.99995	0.99995	0.99995	
Electric rods										[80]
Unit capacity	MW	1.2	1.2	1.2	1.2	1.2	1.2	1.2	1.2	
Capex	€ kW^{-1}	123	114	101	92	83	77	73	71	based on LR
Opex $_{fix}$	% of capex p.a.	1	1	1	1	1	1	1	1	
Opex $_{var}$	€ $kWh^{-1}{}_{th}$	0	0	0	0	0	0	0	0	
Lifetime	year	30	30	30	30	30	30	30	30	
Efficiency	%	99.8	99.8	99.8	99.8	99.8	99.8	99.8	99.8	
Availability	%	100	100	100	100	100	100	100	100	
Very High Temperature (VHT) heat storage in molten material										[80]
Capacity	MWh_{th}	5	6	6	6	6	6	6	6	
Capex	€ $kWh^{-1}{}_{th}$	63	56	46	40	34	31	29	28	
Opex $_{fix}$	% of capex p.a.	1.0	1.0	1.0	1.0	1.0	1.0	1.0	1.0	
Opex $_{var}$	€ $kWh^{-1}{}_{th}$	0.0001	0.0001	0.0001	0.0001	0.0001	0.0001	0.0001	0.0001	
Lifetime	year	30	30	30	30	30	30	30	30	
Charge eff.	%	100	100	100	100	100	100	100	100	
Discharge eff.	%	99.0	99.0	99.0	99.0	99.0	99.0	99.0	99.0	
Self-discharge	% h^{-1}	0.17	0.17	0.17	0.17	0.17	0.17	0.17	0.17	
Availability	%	100	100	100	100	100	100	100	100	
Decoupled steam generator										[80]
Unit capacity	MW_{th}	6	6	8	8	8	8	8	8	
Capex	€ $kW^{-1}{}_{th}$	137	117	91	76	61	53	48	45	
Opex $_{fix}$	% of capex p.a.	3%	3%	3%	3%	3%	3%	3%	3%	
Opex $_{var}$	€ $kWh^{-1}{}_{th}$	0	0	0	0	0	0	0	0	
Lifetime	year	30	30	30	30	30	30	30	30	
Heat to steam eff.	%	99.7	99.7	99.7	99.7	99.7	99.7	99.7	99.7	
Warm water to steam eff.	%	94.8	94.8	94.8	94.8	95.0	95.2	95.4	95.6	
Availability	%	98.1	98.1	98.1	98.1	98.1	98.1	98.1	98.1	
Chiller (10 °C to 4 °C)										[66]
Unit capacity	t_{H2O} h^{-1}	1700	1700	1700	1700	1700	1700	1700	1700	
Capex	€ $t^{-1}{}_{H2O}$ h	1176	1176	1176	1000	1000	1000	1000	1000	
Opex $_{fix}$	% of capex p.a.	4	4	4	3	3	3	3	3	
Opex $_{var}$	€ $t^{-1}{}_{H2O}$	0	0	0	0	0	0	0	0	

Item	Unit	2020/ref.	2025	2028	2030	2035	2040	2045	2050	Ref.
Electricity consumption	kWh t^{-1}_{H2O}	2	2	2	2	2	2	2	2	
Lifetime	year	15	15	15	15	15	15	15	15	
Availability	%	91.3	91.3	91.3	91.3	91.3	91.3	91.3	91.3	
Integrated chilled-cold water tank										[66]
Capacity	t_{H2O}	140 000	140 000	140 000	140 000	140 000	140 000	140 000	140 000	
Capex	€ t^{-1}_{H2O}	143	143	143	143	143	143	143	143	
Opex $_{fix}$	% of capex p.a.	0.7	0.7	0.7	0.7	0.7	0.7	0.7	0.7	
Opex $_{var}$	€ t^{-1}_{H2O}	0.01	0.01	0.01	0.01	0.01	0.01	0.01	0.01	
Lifetime	year	30	30	30	30	30	30	30	30	
Cycle efficiency	%	100	100	100	100	100	100%	10	100	
Energy-to-Power ratio	h	12	12	12	12	12	12	12	12	
Self-discharge	% h^{-1}	0	0	0	0	0	0	0	0	
Single-Cell Protein core plant - Reference scenario										this study
Productivity	g_{CDW} L^{-1}_{WC} h^{-1}			1.0	1.0	1.1	1.2	1.3	1.4	
Unit capacity	t_{SCP} h^{-1}			2.053	20.526	20.526	20.526	20.526	20.526	
	kt_{SCP} a^{-1} – 8000 FLh			16.42	164.2	164.2	164.2	164.2	164.2	
Capex	€ t^{-1}_{SCP} a			8 995	5 341	3 813	3 136	2 654	2 286	
	€ kg^{-1}_{SCP} h			71 960	42 728	30 504	25 088	21 232	18 288	
Opex $_{fix}$	€ t^{-1}_{SCP} a			503	261	193	162	140	123	
	€ kg^{-1}_{SCP} h			4 027	2 086	1 540	1 294	1 118	981	
Opex $_{var}$	€ t^{-1}_{SCP}			75	75	73	70	69	67	
Lifetime	year			25	25	25	25	25	25	
H_2 consumption	kg_{H2} kg^{-1}_{SCP}			0.4281	0.4281	0.4281	0.4281	0.4281	0.4281	
	$kWh_{H2,HHV}$ kg^{-1}_{SCP}			16.872	16.872	16.872	16.872	16.872	16.872	
CO_2 consumption	kg_{CO2} kg^{-1}_{SCP}			1.824	1.824	1.824	1.824	1.824	1.824	
O_2 consumption	kg_{O2} kg^{-1}_{SCP}			1.989	1.989	1.989	1.989	1.989	1.989	
Electricity consumption	kWh_{el} kg^{-1}_{SCP}			8.475	8.475	7.810	7.255	6.786	6.384	
Steam consumption	kWh_{steam} kg^{-1}_{SCP}			5.657	5.657	5.448	5.274	5.126	4.999	
NH_3 consumption	kg_{NH3} kg^{-1}_{SCP}			0.148	0.148	0.148	0.148	0.148	0.148	
Chilled water consumption	kg_{ChW} kg^{-1}_{SCP}			1686	1686	1635	1593	1557	1526	
Heat from dryer water vapour condensing to 70 °C	kWh_{th} kg^{-1}_{SCP}			2.68	2.68	2.68	2.68	2.68	2.68	
Dryer water vapour capture rate	%			50	50	55	60	65	70	
Dryer water vapour's utilisable heat	kWh_{th} kg^{-1}_{SCP}			1.34	1.34	1.474	1.608	1.742	1.876	
Minimum load	% of full capacity			50	50	50	50	50	50	
Ramp-up time	% h^{-1}			1	1	1	1	1	1	
Ramp-down time	% h^{-1}			0.3	0.3	0.3	0.3	0.3	0.3	
Ramp-up/down cost	€ t^{-1}_{SCP} h^2			200	200	200	200	200	200	

Item	Unit	2020/ref.	2025	2028	2030	2035	2040	2045	2050	Ref.
Availability	h			8000	8000	8000	8000	8000	8000	
SCP storage										Solar Foods
Capacity	t_{SCP}			690	690	690	690	690	690	
Capex	€ t^{-1}_{SCP}			350	350	350	350	350	350	
Opex $_{fix}$	% of capex p.a.			1.0%	1.0%	1.0%	1.0%	1.0%	1.0%	
Opex $_{var}$	€ t^{-1}_{SCP}			5	5	5	5	5	5	
Lifetime	year			30	30	30	30	30	30	
Cycle efficiency	%			100	100	100	100	100	100	
Energy-to-Power ratio	h			168	168	168	168	168	168	
Self-discharge	% h^{-1}			0	0	0	0	0	0	
O_2 initial handling (small tank and compressor coupled to 2 stacks) - reference scenario										[66,67]
Tank pressure	bara	5	5	5	5	5	5	5	5	
Capacity	kg_{O2} h^{-1}	692	692	692	692	692	692	692	692	
Capex	€ kg^{-1}_{O2} h	526	526	526	372	316	287	265	246	based on LR
Opex $_{fix}$	% of capex p.a.	3.0	3.0	3.0	3.0	3.0	3.0	3.0	3.0	
Opex $_{var}$	€ t^{-1}_{O2}	0.1	0.1	0.1	0.1	0.1	0.1	0.1	0.1	
Lifetime	year	30	30	30	30	30	30	30	30	
O_2 Compressor										
Pressure range	bara	5→15	5→15	5→15	5→15	5→15	5→15	5→15	5→15	
Electricity consumption	kWh t^{-1}_{O2}	39	39	39	39	39	39	39	39	[22]
Capacity	t_{CO2} h^{-1}	4	4	4	10	10	10	10	10	
	kW_{el}	156	156	156	390	390	390	390	390	
Capex	€ kW^{-1}	11 200	11 200	11 200	5850	5850	5850	5850	5850	[23]
	€ kg^{-1}_{O2} h	437	437	437	228	228	228	228	228	
Opex $_{fix}$	% of capex p.a.	4.0	4.0	4.0	4.0	4.0	4.0	4.0	4.0	
	€ t^{-1}_{O2} a	0.91	0.91	0.91	1.44	1.44	1.44	1.44	1.44	
Opex $_{var}$ - excl. electricity	€ t^{-1}_{O2}	0.1	0.1	0.1	0.1	0.1	0.1	0.1	0.1	
Lifetime	year	20	20	20	20	20	20	20	20	
O_2 liquefaction plant - receiving O_2 at 5 bar										[66]
Electricity consumption	kWh t^{-1}_{O2}	250	250	250	250	250	250	250	250	
Capacity	t_{CO2} h^{-1}	1.50	1.50	1.50	15	15	15	15	15	
	kW_{el}	375	375	375	3750	3750	3750	3750	3750	
Capex	€ kg^{-1}_{O2} h	1000	1000	1000	730	730	730	730	730	
	€ t^{-1}_{O2} a	114	114	114	83	83	83	83	83	
Opex $_{fix}$	% of capex p.a.	3.5	3.5	3.5	3.1	3.1	3.1	3.1	3.1	
Opex $_{var}$ - excl. electricity	€ t^{-1}_{O2}	0.2	0.2	0.2	0.2	0.2	0.2	0.2	0.2	
Lifetime	year	20	20	20	20	20	20	20	20	

Item	Unit	2020/ref.	2025	2028	2030	2035	2040	2045	2050	Ref.
O_2 (g) storage – low pressure storage in spherical tanks										66
Maximum pressure	bar	15	15	15	15	15	15	15	15	
Unit capacity	m^3	4000	4000	4000	4000	4000	4000	4000	4000	
	t_{O2}	73	73	73	73	73	73	73	73	
Capex	€ t^{-1}_{O2}	48 000	48 000	48 000	48 000	48 000	48 000	48 000	48 000	
Opex $_{fix}$	% of capex p.a.	1.5	1.5	1.5	1.5	1.5	1.5	1.5	1.5	
Opex $_{var}$	€ t^{-1}_{O2}	0.1	0.1	0.1	0.1	0.1	0.1	0.1	0.1	
Lifetime	year	30	30	30	30	30	30	30	30	
Cycle eff.	%	99	99	99	99	99	99	99	99	
Self-discharge	% h^{-1}	0.00014	0.00014	0.00014	0.00014	0.00014	0.00014	0.00014	0.00014	
O2 (l) storage tank – standard pressure vessel tanks for liquified oxygen with volumes up to 60 m^3 and liquid oxygen storage for larger capacities										66
Capacity	m^3	60	60	60	600	600	600	600	600	
	t_{O2}	68	68	68	680	680	680	680	680	
Capex	€ t^{-1}_{O2}	5900	5900	5900	4800	4800	4800	4800	4800	
Opex $_{fix}$	% of capex p.a.	1.7	1.7	1.7	1.5	1.5	1.5	1.5	1.5	
Opex $_{var}$	€ t^{-1}_{O2}	0.2	0.2	0.2	0.2	0.2	0.2	0.2	0.2	
Lifetime	year	30	30	30	30	30	30	30	30	
Cycle eff.	%	99	99	99	99	99	99	99	99	
Self-discharge	% h^{-1}	0.001	0.001	0.001	0.001	0.001	0.001	0.001	0.001	

References

1. Food and Agriculture Organization of the United Nations. Food and agriculture projections to 2050. https://www.fao.org/global-perspectives-studies/food-agriculture-projections-to-2050/en (2022).
2. Järviö, N., Maljanen, N. L., Kobayashi, Y., Ryynänen, T. & Tuomisto, H. L. An attributional life cycle assessment of microbial protein production: A case study on using hydrogen-oxidizing bacteria. *Science of the Total Environment* 776, 145764 (2021).
3. Ishizaki, A. & Tanaka, K. Batch culture of Alcaligenes eutrophus ATCC 17697T using recycled gas closed circuit culture system. *J Ferment Bioeng* 69, (1990).
4. Bongers, L. Energy generation and utilization in hydrogen bacteria. *J Bacteriol* 104, 145–151 (1970).
5. García Martínez, J. B. *et al.* Potential of microbial protein from hydrogen for preventing mass starvation in catastrophic scenarios. *Sustain Prod Consum* 25, 234–247 (2021).
6. Jean, A. B. & Brown, R. C. Techno-Economic Analysis of Gas Fermentation for the Production of Single Cell Protein. *Environ Sci Technol* 58, 3823–3829 (2024).
7. Nappa, M. *et al.* Solar-Powered Carbon Fixation for Food and Feed Production Using Microorganisms - A Comparative Techno-Economic Analysis. *ACS Omega* 5, 33242–33252 (2020).
8. Pikaar, I. *et al.* Decoupling Livestock from Land Use through Industrial Feed Production Pathways. *Environ Sci Technol* 52, 7351–7359 (2018).
9. Klinzing, K. *et al.* In vitro genotoxicological evaluation of protein-rich powder derived from *Xanthobacter* sp. *SoF1*. *Journal of Applied Toxicology* 1–14 (2024) doi:10.1002/jat.4621.
10. ’Holmström, S. & ’Pitkänen, J.-P. Strains and processes for single cell protein or biomass production. (2021).
11. Liu, C., Colón, B. C., Ziesack, M., Silver, P. A. & Nocera, D. G. Water splitting-biosynthetic system with CO2 reduction efficiencies exceeding photosynthesis. *Science (1979)* 352, (2016).
12. Sillman, J. *et al.* Bacterial protein for food and feed generated via renewable energy and direct air capture of CO2: Can it reduce land and water use? *Glob Food Sec* 22, 25–32 (2019).
13. Sillman, J. *et al.* A life cycle environmental sustainability analysis of microbial protein production via power-to-food approaches. *International Journal of Life Cycle Assessment* 25, 2190–2203 (2020).
14. Leger, D. *et al.* Photovoltaic-driven microbial protein production can use land and sunlight more efficiently than conventional crops. *Proc Natl Acad Sci U S A* 118, No. 26 e2015025118 (2021).
15. Daneels, R. *Power to Gas to Protein - Protein at Farm Scale from Feed Compatible Components*. (2015).
16. Matassa, S., Verstraete, W., Pikaar, I. & Boon, N. Autotrophic nitrogen assimilation and carbon capture for microbial protein production by a novel enrichment of hydrogen-oxidizing bacteria. *Water Res* 101, (2016).
17. Liu, C., Colón, B. C., Ziesack, M., Silver, P. A. & Nocera, D. G. Water splitting-biosynthetic system with CO2 reduction efficiencies exceeding photosynthesis. *Science (1979)* 352, 1210–1213 (2016).
18. KWR. *Power-to-Protein: Eiwitproductie in Een Circulaire Economie - Fase 2 Pilotonderzoek*. https://edepot.wur.nl/474660 (2019).
19. Aidoo, R., Kwofie, E. M., Adewale, P., Lam, E. & Ngadi, M. Overview of single cell protein: Production pathway, sustainability outlook, and digital twin potentials. *Trends in Food Science and Technology* vol. 138 Preprint at https://doi.org/10.1016/j.tifs.2023.07.003 (2023).

20. Jørgensen, J. B. Exploiting Microorganisms for Animal Feed Production. in *The 7th Malaysia International Agro-Bio Business Conference: What Next Agriculture II – Another Food Crisis?* (Kuala Lumpur, 2011).

21. Sunfire. Sunfire-HyLink alkaline electrolyzer factsheet. Preprint at https://www.sunfire.de/files/sunfire/images/content/Produkte_Technologie/factsheets/Sunfire-Factsheet-HyLink-Alkaline_202405.pdf (2024).

22. Aspen Technology. *Aspen Plus*. (2024).

23. Towler, G. & Sinnott, R. *Chemical Engineering Design: Principles, Practice and Economics of Plant and Process Design*. *Chemical Engineering Design: Principles, Practice and Economics of Plant and Process Design* (Butterworth-Heinemann, Oxford, 2021). doi:10.1016/B978-0-12-821179-3.01001-3.

24. Junginger, M. & Louwen, A. *Technological Learning in the Transition to a Low-Carbon Energy System: Conceptual Issues, Empirical Findings, and Use in Energy Modeling*. *Technological Learning in the Transition to a Low-Carbon Energy System: Conceptual Issues, Empirical Findings, and Use, in Energy Modeling* (Academic Press, London, 2019). doi:10.1016/C2018-0-04547-8.

25. Schmidt, O. *et al.* Future cost and performance of water electrolysis: An expert elicitation study. *Int J Hydrogen Energy* 42, 30470–30492 (2017).

26. Schoots, K., Ferioli, F., Kramer, G. J. & van der Zwaan, B. C. C. Learning curves for hydrogen production technology: An assessment of observed cost reductions. *Int J Hydrogen Energy* 33, 2630–2645 (2008).

27. Rogner, H. H. Hydrogen technologies and the technology learning curve. *Int J Hydrogen Energy* 23, 833–840 (1998).

28. Bogdanov, D. *et al.* Low-cost renewable electricity as the key driver of the global energy transition towards sustainability. *Energy* 227, 120467 (2021).

29. Galimova, T. *et al.* Global trading of renewable electricity-based fuels and chemicals to enhance the energy transition across all sectors towards sustainability. *Renewable and Sustainable Energy Reviews* 183, 113420 (2023).

30. IEA. *Global Hydrogen Review 2023*. https://www.iea.org/reports/global-hydrogen-review-2023 (2023).

31. Saba, S. M., Müller, M., Robinius, M. & Stolten, D. The investment costs of electrolysis – A comparison of cost studies from the past 30 years. *International Journal of Hydrogen Energy* vol. 43 Preprint at https://doi.org/10.1016/j.ijhydene.2017.11.115 (2018).

32. Proost, J. State-of-the art CAPEX data for water electrolysers, and their impact on renewable hydrogen price settings. *Int J Hydrogen Energy* (2019) doi:10.1016/j.ijhydene.2018.07.164.

33. NEL. Press releases. *NEL Hydrogen* https://nelhydrogen.com/press-releases/ (2023).

34. NEL. *Fourth Quarter 2022 Results Presentation*. https://nelhydrogen.com/wp-content/uploads/2023/02/Q4-report-2022-FOR-PUBLICATION.pdf (2023).

35. HydrogenInsight. Auction results reveal that Chinese hydrogen electrolysers are two to five times cheaper to buy than Western machines. *HydrogenInsight* https://www.hydrogeninsight.com/electrolysers/auction-results-reveal-that-chinese-hydrogen-electrolysers-are-two-to-five-times-cheaper-to-buy-than-western-machines/2-1-1570717 (2023).

36. BNEF. *Electrolyzer Price Survey 2024: Rising Costs, Glitchy Tech*. (2024).

37. BloombergNEF. 2023 Hydrogen Levelized Cost Update: Green Beats Gray. *Bloomberg New Energy Finance* https://about.bnef.com/blog/2023-hydrogen-levelized-cost-update-green-beats-gray/ (2023).

38. Krishnan, S. *et al.* Present and future cost of alkaline and PEM electrolyser stacks. *Int J Hydrogen Energy* 48, (2023).

39. Glenk, G. & Reichelstein, S. Economics of converting renewable power to hydrogen. *Nat Energy* 4, 216–222 (2019).

40. IRENA. *Green Hydrogen Cost Reduction: Scaling up Electrolysers to Meet the 1.50C Climate Goal*. *IRENA* www.irena.org/publications (2020).

41. Climeworks. *Climeworks DACS Factsheet for Researchers*. http://www.climeworks.com/wp-content/uploads/2018/10/DACS-Factsheet-for-researchers.pdf (2019).

42. Beuttler, C., Charles, L. & Wurzbacher, J. The Role of Direct Air Capture in Mitigation of Anthropogenic Greenhouse Gas Emissions. *Frontiers in Climate* 1, 10 (2019).

43. Climeworks. Articles and FAQ about direct air capture. How much energy is required to run Climeworks' direct air capture process? https://web.archive.org/web/20210927092014/https:/climeworks.com/faq-about-direct-air-capture (2021).

44. Deutz, S. & Bardow, A. Life-cycle assessment of an industrial direct air capture process based on temperature–vacuum swing adsorption. *Nat Energy* 6, 203–213 (2021).

45. 'Wurzbacher, J. & 'Gebald, C. Climeworks opening presentation at Direct Air Capture Summit. *Climeworks 4th Direct Air Capture Summit* Preprint at (2023).

46. National Academies of Sciences Engineering and Medicine. *Negative Emissions Technologies and Reliable Sequestration: A Research Agenda*. *National Academies Press* (The National Academies Press, Washington, D.C., 2019). doi:https://doi.org/10.17226/25259.

47. Sabatino, F. *et al.* A comparative energy and costs assessment and optimization for direct air capture technologies. *Joule* 5, 2047–2076 (2021).

48. Sendi, M., Bui, M., Mac Dowell, N. & Fennell, P. Geospatial analysis of regional climate impacts to accelerate cost-efficient direct air capture deployment. *One Earth* 5, 1153–1164 (2022).

49. Wiegner, J. F., Grimm, A., Weimann, L. & Gazzani, M. Optimal Design and Operation of Solid Sorbent Direct Air Capture Processes at Varying Ambient Conditions. *Ind Eng Chem Res* 61, 12649–12667 (2022).

50. Bloomberg. World's Largest Carbon-Sucking Plant Starts Making Tiny Dent in Emissions. *Bloomberg* https://www.bloomberg.com/news/features/2021-09-08/inside-the-world-s-largest-direct-carbon-capture-plant (2021).

51. IEA. CCUS Projects Database. *International Energy Agency* https://www.iea.org/data-and-statistics/data-product/ccus-projects-database (2023).

52. Fasihi, M., Efimova, O. & Breyer, C. Techno-economic assessment of CO2 direct air capture plants. *J Clean Prod* 224, 957–980 (2019).

53. Young, J. *et al.* The cost of direct air capture and storage can be reduced via strategic deployment but is unlikely to fall below stated cost targets. *One Earth* 6, 899–917 (2023).

54. Aghahosseini, A. & Breyer, C. Assessment of geological resource potential for compressed air energy storage in global electricity supply. *Energy Convers Manag* 169, 161–173 (2018).

55. ETIP-PV. *The True Competitiveness of Solar PV. A European Case Study*. https://goo.gl/FBzSJx (2017).

56. Vartiainen, E., Masson, G., Breyer, C., Moser, D. & Román Medina, E. Impact of weighted average cost of capital, capital expenditure, and other parameters on future utility-scale PV levelised cost of electricity. *Progress in Photovoltaics: Research and Applications* 28, 439–453 (2020).

57. Bolinger, M. & Bolinger, G. Land Requirements for Utility-Scale PV: An Empirical Update on Power and Energy Density. *IEEE J Photovolt* 12, 589–594 (2022).

58. Bogdanov, D. *et al.* Radical transformation pathway towards sustainable electricity via evolutionary steps. *Nat Commun* 10, 1077 (2019).

59. Bolinger, M., Seel, J. & Hamachi LaCommare, K. *Utility-Scale Solar 2016: An Empirical Analysis of Project Cost, Performance, and Pricing Trends in the United States, Lawrence Berkeley National Laboratory*. https://emp.lbl.gov/sites/default/files/utility-scale-solar-2016-report.pdf (2017).

60. [EC] - European Commission. *Technology Pathways in Decarbonisation Scenarios*. *EU-ASSET Project Deliverable* https://op.europa.eu/en/publication-detail/-/publication/599a1d8e-509a-11eb-b59f-01aa75ed71a1/language-en (2018).

61. Bogdanov, D. & Breyer, C. North-East Asian Super Grid for 100% renewable energy supply: Optimal mix of energy technologies for electricity, gas and heat supply options. *Energy Convers Manag* 112, 176–190 (2016).

62. Neij, L. Cost development of future technologies for power generation — A study based on experience curves and complementary bottom-up assessments. *Energy Policy* 36, 2200–2211 (2008).

63. Breyer, C. *et al.* Solar photovoltaics demand for the global energy transition in the power sector. *Progress in Photovoltaics: Research and Applications* 26, 505–523 (2018).

64. [EC] - European Commission. *ETRI 2014: Energy Technology Reference Indicator Projections for 2010-2050; Joint Research Centre (JRC), European Commission (EC). Publication Office of the European Union* (Luxembourg, 2014).

65. Farfan, J. & Breyer, C. Structural changes of global power generation capacity towards sustainability and the risk of stranded investments supported by a sustainability indicator. *J Clean Prod* 141, 370–384 (2017).

66. Sweco Finland Holding Oy. *Cost Estimates for Green Field Power to Food Plant Utilities and Storage Systems - Internal Report for Solar Foods Oy*. (2019).

67. Confidential. *Confidential Budget Quote from a European Electrolyser Supplier*. (2020).

68. Makridis, S. S. Hydrogen storage and compression. in *Methane and Hydrogen for Energy Storage* (eds. Carriveau, R. & Ting, D. S.-K.) 1–28 (IET Digital Library, 2016).

69. Papadias, D. D. & Ahluwalia, R. K. Bulk storage of hydrogen. *Int J Hydrogen Energy* 46, 34527–34541 (2021).

70. DNV KEMA. *Systems Analyses Power to Gas a Technology Review | Energy Storage | Wind Power | Free 30-Day Trial | Scribd*. https://www.scribd.com/document/328966622/DNV-KEMA-Systems-Analyses-Power-to-Gas-a-Technology-Review (2013).

71. Fasihi, M. & Breyer, C. Baseload electricity and hydrogen supply based on hybrid PV-wind power plants. *J Clean Prod* 243, 118466 (2020).

72. Michalski, J. *et al.* Hydrogen generation by electrolysis and storage in salt caverns: Potentials, economics and systems aspects with regard to the German energy transition. *Int J Hydrogen Energy* 42, 13427–13443 (2017).

73. Crotogino, F., Donadei, S., Bünger, U. & Landinger, H. Large-Scale Hydrogen Underground Storage for Securing Future Energy Supplies. *18th World Hydrogen Energy Conference 2010 - WHEC 2010 Parallel Sessions Book 4: Storage Systems / Policy Perspectives, Initiatives and Co-operations* 78, (2010).

74. Fasihi, M., Weiss, R., Savolainen, J. & Breyer, C. Global potential of green ammonia based on hybrid PV-wind power plants. *Appl Energy* 294, 116170 (2021).

75. ISPT. *Power to Ammonia*. *Institute for Sustainable Process Technology* (2017).

76. Morgan, E. R. Techno-economic feasibility study of ammonia plants powered by offshore wind. *University of Massachusetts - Amherst, PhD Dissertations* (University of Massachusetts Amherst, Amherst, 2013).

77. Danish Energy Agency. *Technology Data for Industrial Process Heat - Version 4*. https://ens.dk/en/our-services/technology-catalogues/technology-data-industrial-process-heat (2022).

78. Christidis, A. C. Thermische Speicher zur Optimierung des Betriebs von Heizkraftwerken in der Fernwärmeversorgung. *Technische Universität Berlin* (Technical University of Berlin, Berlin, 2019).

79. Kraft, A. Personal communication on hot water storage tank. *EEB ENERKO Energiewirtschaftliche Beratung GmbH* Preprint at (2020).

80. Elstor Oy. Potential development of Elstor technology. Preprint at https://elstor.fi/en/technology-and-services/ (2022).
